# Ranking and Rating Bicycle Helmet Safety Performance in Oblique Impacts Using Eight Different Brain Injury Models

**DOI:** 10.1007/s10439-020-02703-w

**Published:** 2021-01-21

**Authors:** Madelen Fahlstedt, Fady Abayazid, Matthew B. Panzer, Antonia Trotta, Wei Zhao, Mazdak Ghajari, Michael D. Gilchrist, Songbai Ji, Svein Kleiven, Xiaogai Li, Aisling Ní Annaidh, Peter Halldin

**Affiliations:** 1 Division of Neuronic Engineering, KTH Royal Institute of Technology, Hälsovägen 11C, 141 52 Huddinge, Sweden; 2 Dyson School of Design Engineering, Imperial College London, London, UK; 3 Department of Mechanical and Aerospace Engineering, University of Virginia, Charlottesville, VA USA; 4 Department of Biomedical Engineering, University of Virginia, Charlottesville, VA USA; 5 School of Mechanical & Materials Engineering, University College Dublin, Belfield, Dublin 4, Ireland; 6 Department of Biomedical Engineering, Worcester Polytechnic Institute, Worcester, MA 01605 USA; 7 Department of Mechanical Engineering, Worcester Polytechnic Institute, Worcester, MA 01609 USA; 8 School of Medicine and Medical Science, University College Dublin, UCD Charles Institute of Dermatology, Belfield, Dublin 4, Ireland

**Keywords:** Bicycle helmet, Brain injury criteria, Concussion, Finite element models, Oblique impact tests, Test methods

## Abstract

Bicycle helmets are shown to offer protection against head injuries. Rating methods and test standards are used to evaluate different helmet designs and safety performance. Both strain-based injury criteria obtained from finite element brain injury models and metrics derived from global kinematic responses can be used to evaluate helmet safety performance. Little is known about how different injury models or injury metrics would rank and rate different helmets. The objective of this study was to determine how eight brain models and eight metrics based on global kinematics rank and rate a large number of bicycle helmets (n=17) subjected to oblique impacts. The results showed that the ranking and rating are influenced by the choice of model and metric. Kendall’s tau varied between 0.50 and 0.95 when the ranking was based on maximum principal strain from brain models. One specific helmet was rated as 2-star when using one brain model but as 4-star by another model. This could cause confusion for consumers rather than inform them of the relative safety performance of a helmet. Therefore, we suggest that the biomechanics community should create a norm or recommendation for future ranking and rating methods.

**Electronic supplementary material:**

The online version of this article (10.1007/s10439-020-02703-w) contains supplementary material, which is available to authorized users.

## Introduction

Head injuries are a significant problem in society that can cause both acute and long-term consequences. The head is also the most common body region for severe injuries among bicyclists.^41^ Bicycle helmets have been shown to mitigate the severity of injury during head impacts by reducing the forces acting on the head.^8^,^9^,^15^,^37^ In most countries, helmets need to pass a specific certification standard to be allowed into the market. Today, most helmet test standards evaluate a helmet’s safety performance using only the measured linear acceleration of a dummy headform resulting from impacts against a rigid surface, e.g., a flat surface or a curbstone. In the current bicycle helmet standards,^1^,^7^,^13^ the pass/fail threshold for a helmet ranges between 250 and 300 g peak linear acceleration, depending on the standard.

Since the 1940 s, research has demonstrated that the mechanisms associated with diffuse-type brain injuries are more sensitive to rotational motion of the head compared to linear motion especially for diffuse brain injuries.^28^ Despite this, rotational head motion is not reflected in the current bicycle helmet test standards, although there is ongoing work within the European standards organization (CEN/TC158) to develop a new test method for helmets. The new standard will include rotational measures of head response and will include oblique impacts that often result in substantial rotational head motion.

While oblique impacts have not been formally adopted into the test standards, several studies have used these conditions to rate different helmet designs.^4^,^10^,^11^,^47^ Bland *et al.*^4^ evaluated and rated bicycle helmets by using a metric that combines peak resultant linear head acceleration and the resultant change in angular head velocity. Both Deck *et al.*^11^ and Stigson *et al.*^47^ evaluated and rated helmets using a metric based on the intracranial response of a finite element (FE) brain model (using the Strasbourg Finite Element Head Model SUFEHM and the KTH Royal Institute of Technology head model, respectively). In those studies, linear and angular head kinematics measured during the helmet impact tests were applied to the FE brain models. Then, the resulting deformation of the brain tissue was used to evaluate the performance of the helmets. The methodology for assessing brain injury using output from a dummy headform as input to FE brain models has become common practice in many different areas of safety research, including their use in automotive crash (e.g., Gabler *et al.*^18^) and sports helmet assessment (e.g., Elkin *et al.*^12^ and Clark *et al.*^6^).

More than a dozen different FE brain models have been developed for brain injury research, all with varying levels of anatomical detail, material properties, and boundary conditions between different anatomical regions of the brain. A comprehensive summary of most of these models and their methods is found in a previous publication presented by Giudice *et al.*^27^

Several previous studies have compared different brain injury models and their responses under the same impacts. Baeck^3^ compared three different brain injury models (KTH, SUFEHM and University College Dublin brain trauma model (UCDBTM)) in different fall situations, and concluded that significant disparities were found in the intracranial responses of each model for the same impact conditions. Ji *et al.*^29^ compared three other brain injury models (Dartmouth scaled and normalized model (DSNM), Wayne State University head injury model (WSUHIM), and Simulated Injury Monitor (SIMon) head model) in loading cases from male collegiate hockey games, and found significant disparities in the magnitude and distribution of the brain tissue strain among the three models. In the context of using FE models to assess helmet performance, both studies suggest that it could be possible for helmets to be rated differently depending on which FE model is used.

To introduce an updated helmet test standard, which includes oblique impacts, a relevant pass/fail criterion needs to be established. There is the potential to use tissue-based criteria obtained from FE brain models, such as peak brain tissue strain, as a metric for helmet assessment. There is also the potential to use metrics derived from global kinematic responses, such as change in angular velocity. From previous studies, it is known that there are differences in the results predicted using different FE brain models as mentioned above. However, little is known about how different FE brain models would rank and rate different helmets in realistic impact situations with duration of 10–15 ms, similar to an impact to a road surface.

The objective of this present study was to determine the influence of helmet ranking and rating using a number of existing brain injury models and a variety of existing injury metrics based on global kinematics for bicycle helmets tested in oblique impact conditions against a hard surface. Eight different FE brain models and eight different kinematic-based metrics were included in the comparison.

## Materials and Methods

Throughout this study, the term ‘ranking’ is used to describe the individual position of the helmet amongst the sample tested when organized based on the assessment metric. The term ‘rating’ is used to describe the category to which a helmet belongs when the helmets are clustered into different groups depending on the assessment metric.

The response from eight different FE brain models and eight different well-established kinematic-based metrics were calculated based on the output from the same experimental oblique helmet tests. The results were used to rate and rank the helmets.

### Experimental Data

Experimental tests presented in a previous study^47^ were used in this study to evaluate the influence of the different FE models and kinematic-based metrics on bicycle helmet ranking and rating. The experimental tests included seventeen different conventional bicycle helmets from the Swedish market (2015) (Helmet A to Q). The helmet design varied between the included helmets with twelve street/commuter helmets, three mountain bike helmets, and two skate helmets.

**Figure 1:**
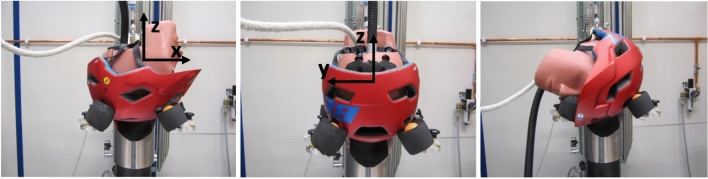
The test setup, from left to right Xrot, Yrot and Zrot together with the anatomical coordinate system of the head.

The helmets were tested in three different impact situations, which caused mainly rotation around the three different anatomical axes of the head (Xrot, Yrot, and Zrot) (Figure 1). Each helmet was tested once for each impact situation. In total, 51 different tests were performed.

The helmeted headforms were dropped onto 45° angled robust steel anvil covered with 80 grit abrasive paper at an impact velocity of 6 m/s. The linear and angular accelerations of the center of gravity of the headform were measured in the head-fixed coordinate system. The kinematic time-histories for all the tests are presented in the supplementary materials.

### Assessment Models

The kinematics from the experiments were applied to the different brain models. The six components of the linear and the angular accelerations were prescribed to the rigid skull at the head center of gravity of each model. All simulations started immediately prior to impact and then ended after 30 ms. The kinematics were filtered before being applied to various brain models. The linear accelerations were filtered with a 1000 cut-off frequency filter, and the angular head accelerations were filtered with an SAE 180 filter.

In total eight different brain models were used in this study, which gave total 408 simulations. The eight different brain models used in this study were:

- Global Human Body Model Consortium (GHBMC) M50-O v4.3^36^
- Imperial College model (IC)^24^
- The isotropic version of KTH model^33^
- PIPER 18 year-old model^35^
- SIMon model^49^
- Total Human Model for Safety (THUMS) v.4.02^2^
- UCDBTM v2.0^50^
- The isotropic version of the Worcester Head Injury Model (WHIM) v.1.0^30^,^54^

All eight models have been developed separately and with different strategies. A short summary of the models is presented in Table 1. Side views of the brain for the different models are shown in the supplementary materials. Previous studies of the brain injury models have used different tissue-response metrics to evaluate the effect on the risk of brain injury from head impacts. In this study, the metrics presented by the respective model developers were used (Table 2). In general, the injury prediction of FE models was based on Maximum Principle Strain (MPS) or the Cumulative Strain Damage Metric (CSDM). In most models, the 95th percentile value of MPS (by element or volume) was taken as the tissue-response to avoid potential numerical issues associated with using the 100th percentile value (which can come from a single element). CSDM is the cumulative volume fraction of elements with MPS exceeding a predefined strain threshold.^49^ In this study, a strain threshold of 0.25 was used for CSDM, which has been used in previous studies.^12^,^48^,^49^ In all models, either true strain or Green-Lagrange (G-L) strain was used. Since this is a study that evaluates existing brain models, the metrics suggested by the developers were used, and therefore different metrics are used across the different models.

**Table 1:** Description of the different brain injury models.

Brain model	Number of brain elements	Number of brain nodes	Intracranial volume [dm^3^]	Element formulation	Brain material properties	Interactions between the different parts of the brain and the brain-skull interface	Software
GHBMC	121.0 k	101.4 k	1.4	Eight-node brick element with constant stress	Linear viscoelastic (standard linear solid)	Tied between brain and falx/tentorium. Continuous mesh brain and subdural CSF.	LS Dyna
IC	386.0 k	576.8 k	1.4	Eight-node brick element with constant stress	Hyperelastic and viscoelastic (Ogden model with Prony series viscoelasticity)	Continuous mesh	LS Dyna
KTH	4.1 k	5.2 k	1.4	Eight-node brick element with selectively reduced integration	Hyperelastic and viscoelastic (Ogden model with Prony series viscoelasticity)	Tangential sliding without separation in normal direction between subdural CSF and brain and between the CSF located on both sides of the falx and tentorium and the brain.	LS Dyna
PIPER	14.4 k	15.9 k	2.2	Eight-node brick element with constant stress	Hyperelastic and viscoelastic (Ogden model with Prony series viscoelasticity)	Continuous mesh for the brain. Tied contact between skull and dura mater.	LS Dyna
SIMon	42.1 k	42.5 k	1.4	Eight-node brick element with constant stress	Linear viscoelastic (standard linear solid)	Continuous mesh	LS Dyna
THUMS	118.8 k	128.2 k	1.6	Eight-node brick element with constant stress	Linear viscoelastic (standard linear solid)	Automatic surface to surface between pia sagittal and falx. Continuous mesh brain and subdural CSF.	LS Dyna
UCDBTM	56.6 k	62.2 k	1.4	Eight-node brick element with reduced integration	Hyperelastic and viscoelastic (Neo-hookean model with Prony series viscoelasticity)	Tangential sliding without separation in normal direction between brain and pia, falx and tentorium	Abaqus
WHIM	55.1 k	56.6 k	1.5	Eight-node brick element with reduced integration	Hyperelastic and viscoelastic (Ogden model with Prony series viscoelasticity)	Continuous mesh	Abaqus

**Table 2:** Outputs from the models used in this study and previous publications of the model developers.

Brain model	Metrics used by the developers
GHBMC	95th percentile true strain and CSDM
IC	90th percentile G-L strain and G-L strain rate
KTH	100th percentile G-L strain
PIPER	95th percentile G-L strain
SIMon	95th percentile true strain and CSDM
THUMS	95th percentile G-L strain
UCDBTM	100th percentile G-L strain
WHIM	95th percentile G-L strain

The ranking and rating of the different brain models were also compared to metrics based on the global kinematics: peak resultant linear acceleration (PLA), peak angular acceleration (PAA), change in resultant angular velocity (PAV), Brain Injury Criterion (BrIC),^48^ Universal Brain Injury Criterion (UBrIC),^19^ Head Injury Criterion (HIC)^51^ and Diffuse Axonal, Multi-Axis, General Evaluation (DAMAGE).^20^ Also, a variant of the metric presented by Bland *et al.*^4^ used in the Virginia Tech STAR rating was included as a kinematic-based metric. The STAR score is based on six different impact locations using two different velocities. In the present study, only three different impact locations, slightly different from the impact locations included in the STAR rating, and only one impact velocity was considered. Therefore, a modified STAR score, called STAR*, was calculated (Equation 1), where L stands for the number of impacts, α for PLA, and ω for peak change in angular velocity.

1 $$STAR^{*} = \mathop \sum \limits_{L = 1}^{3} \frac{1}{{1 + e^{{ - \left( { - 10.2 + 0.0433*a + 0.19686 - 0.0002075*a*\omega } \right)}} }}$$

### Data Analysis

The linear correlation between the peak magnitudes of MPS of the models was evaluated with Pearson’s correlation coefficient of determination (*r*^2^). For the nonparametric data, the ranking of the helmets (from 1 to 17) based on the performance of the helmets, the Kendall’s tau 31 was evaluated. The statistical analysis was performed in MATLAB (version 2019a, The MathWorks, Inc., Natick, Massachusetts, United States).

Rating of helmets has previously been presented by giving the helmets different numbers of stars or similar.^4^,^11^,^46^ Deck *et al.*^11^ used the SUFEHM and the corresponding injury risk curve to evaluate the helmet performance from 1 star to 5 stars. A similar method has been applied by Stigson^46^ for the KTH model. Injury risk functions were not available for all eight models included in this study. Therefore, the rating of helmets was made by taking the average value of the intracranial response or kinematic output of the three loading conditions (Xrot, Yrot, and Zrot). Helmets were then graded from 1-star (highest average value of injury metrics) to 4-star (lowest average value of injury metrics) based on the percentile value of the average values for all seventeen helmets. The helmets with an average value of the injury metrics below the 25th percentile value earned 4-star, which was indicative of the best safety performance. Helmets with an average value of the injury metrics between the 25th and 50th percentile value earned a 3-star rating, while those between the 50th and 75th percentile earned 2-star. Helmets that had an average value above the 75th percentile value earned 1-star, which was indicative of the worst safety performance.

## Results

**Figure 2:**
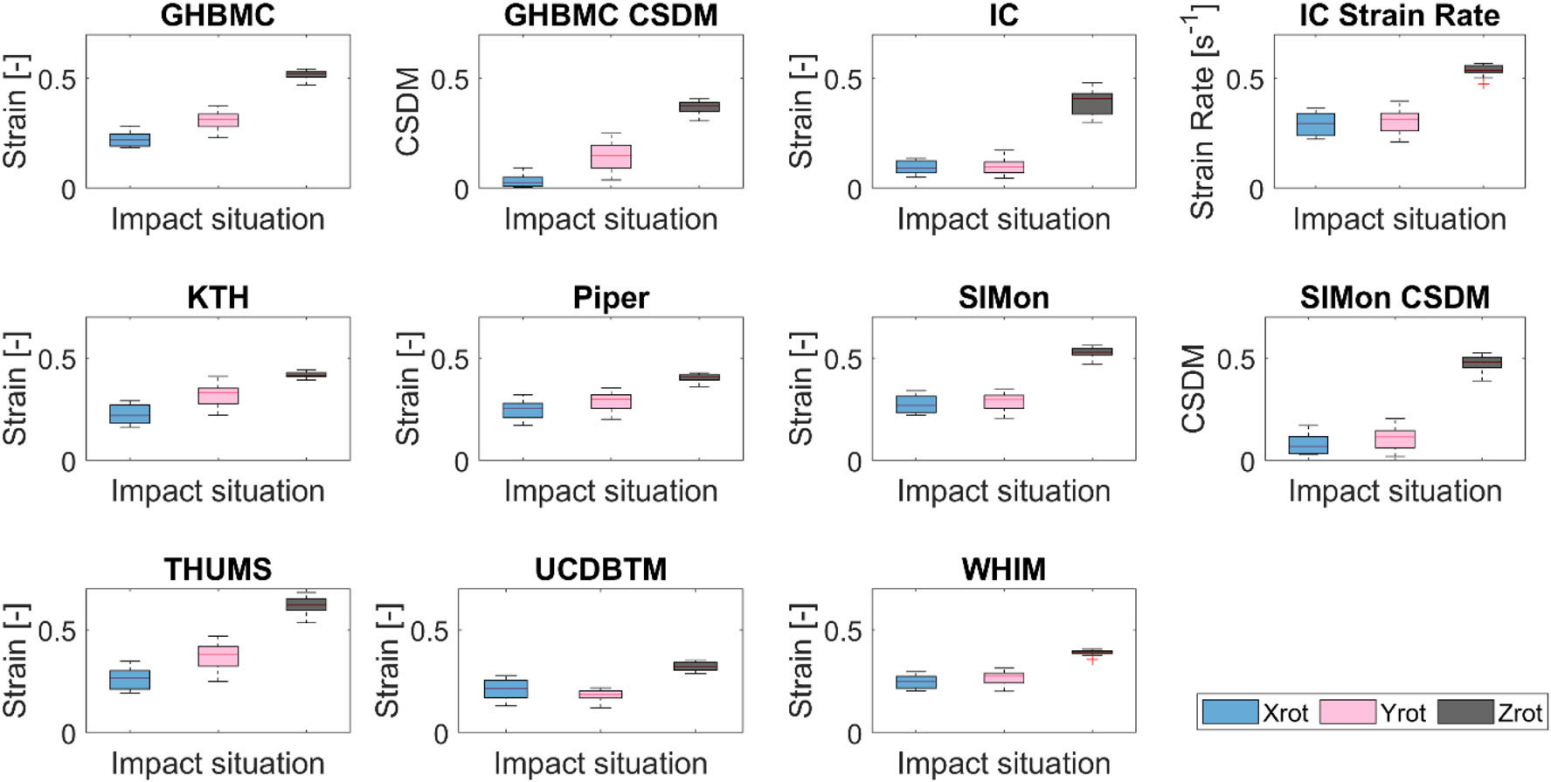
Boxplot of the different models for the three loading conditions based on seventeen different pulses per loading condition.

**Figure 3:**
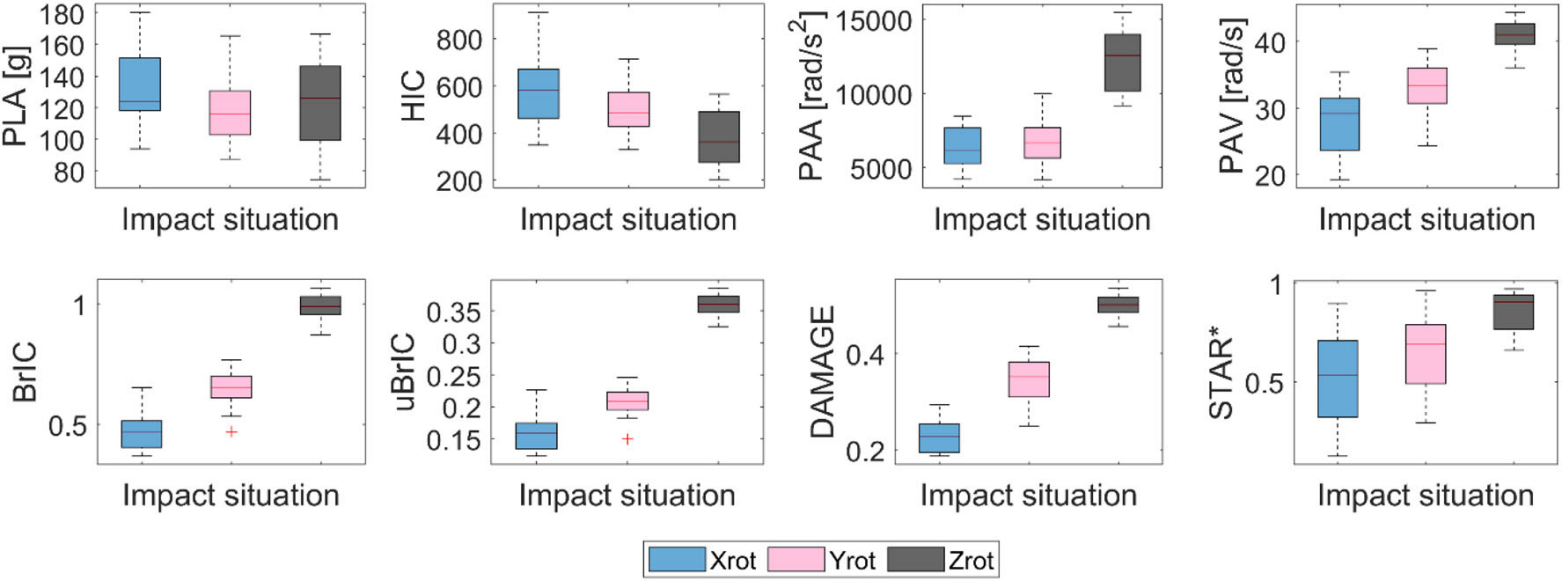
Boxplot of the different kinematic-based metrics for the three loading conditions with seventeen pulses per loading conditions.

A variation of MPS and CSDM is seen between the different models (Figure 2). The response of the models shows higher values for the Zrot compared to Yrot and Xrot. The same trend is observed for the kinematic response based on rotation (Figure 3).

### Linear Correlation

Pearson’s correlation coefficient of determination (*r*^2^) varied between 0.53 and 0.99 between the injury metrics for the different models (Table 3). In general, most brain models correlated well with each other (*r*^2^ > 0.8). The *r*^2^ for each loading condition can be found in the supplementary material.

**Table 3:** Kendall’s tau in the lower triangle and the Pearson’s correlation coefficient of determination (*r*^2^) in the upper triangle.

	GHBMC	GHBMC CSDM	IC	IC Strain Rate	KTH	PIPER	SIMon	SIMon CSDM	THUMS	UCDBTM	WHIM
GHBMC	**–**	**0.990**	**0.878**	**0.924**	**0.899**	**0.933**	**0.937**	**0.949**	**0.990**	0.664	**0.953**
GHBMC CSDM	**0.979**	**–**	**0.841**	**0.899**	**0.914**	**0.933**	**0.908**	**0.922**	**0.990**	0.612	**0.933**
IC	0.648	0.635	**–**	**0.943**	0.691	**0.817**	**0.943**	**0.947**	**0.863**	**0.832**	**0.910**
IC Strain Rate	0.760	0.749	**0.838**	**–**	**0.808**	**0.941**	**0.986**	**0.970**	**0.929**	**0.826**	**0.990**
KTH	**0.905**	**0.889**	0.699	0.779	**–**	**0.920**	0.776	0.769	**0.916**	0.533	**0.854**
PIPER	**0.887**	**0.882**	0.708	**0.851**	**0.868**	**–**	**0.916**	**0.899**	**0.962**	0.689	**0.968**
SIMon	0.787	0.788	0.775	**0.902**	0.767	**0.871**	**–**	**0.994**	**0.935**	**0.805**	**0.984**
SIMon CSDM	**0.838**	**0.842**	0.743	**0.860**	**0.802**	**0.903**	**0.940**	**–**	**0.941**	0.776	**0.970**
THUMS	**0.945**	**0.933**	0.655	0.766	**0.903**	**0.906**	**0.808**	**0.856**	**–**	0.661	**0.958**
UCDBTM	0.513	0.497	0.752	0.691	0.556	0.581	0.650	0.609	0.537	**–**	0.778
WHIM	**0.830**	**0.827**	0.757	**0.915**	**0.823**	**0.923**	**0.920**	**0.920**	**0.838**	0.619	**–**

Highlighted in bold above 0.80

None of the brain models showed linear correlation to the kinematic-based metrics based on linear acceleration (PLA and HIC) or a combination of linear acceleration and angular velocity (STAR*) (Table 4). However, most brain models’ injury metrics had an *r*^2^ above 0.8 for the kinematic-based metrics that were based on angular motion.

**Table 4:** Pearson’s correlation coefficient of determination (*r*^2^) for the different models and the different kinematic-based metrics.

	PLA	HIC	PAA	PAV	BrIC	UBrIC	DAMAGE	STAR*
GHBMC	0.028	0.298	0.734	**0.863**	**0.984**	**0.982**	**0.972**	0.473
GHBMC CSDM	0.030	0.295	0.707	**0.874**	**0.976**	**0.960**	**0.982**	0.475
IC	0.000	0.177	**0.874**	0.679	**0.837**	**0.887**	0.774	0.465
IC Strain Rate	0.007	0.202	**0.828**	**0.830**	**0.904**	**0.931**	**0.850**	0.537
KTH	0.018	0.211	0.664	**0.924**	**0.910**	**0.848**	**0.951**	0.564
PIPER	0.026	0.236	0.729	**0.958**	**0.941**	**0.918**	**0.929**	0.564
SIMon	0.018	0.253	0.781	**0.803**	**0.916**	**0.953**	**0.854**	0.468
SIMon CSDM	0.020	0.274	0.769	0.781	**0.920**	**0.960**	**0.865**	0.440
THUMS	0.031	0.292	0.726	**0.904**	**0.984**	**0.972**	**0.978**	0.497
UCDBTM	0.002	0.100	0.783	0.563	0.634	0.686	0.558	0.448
WHIM	0.018	0.238	0.785	**0.878**	**0.941**	**0.955**	**0.897**	0.527

Highlighted in bold above 0.80

### Correlation of Ranking

**Figure 4:**
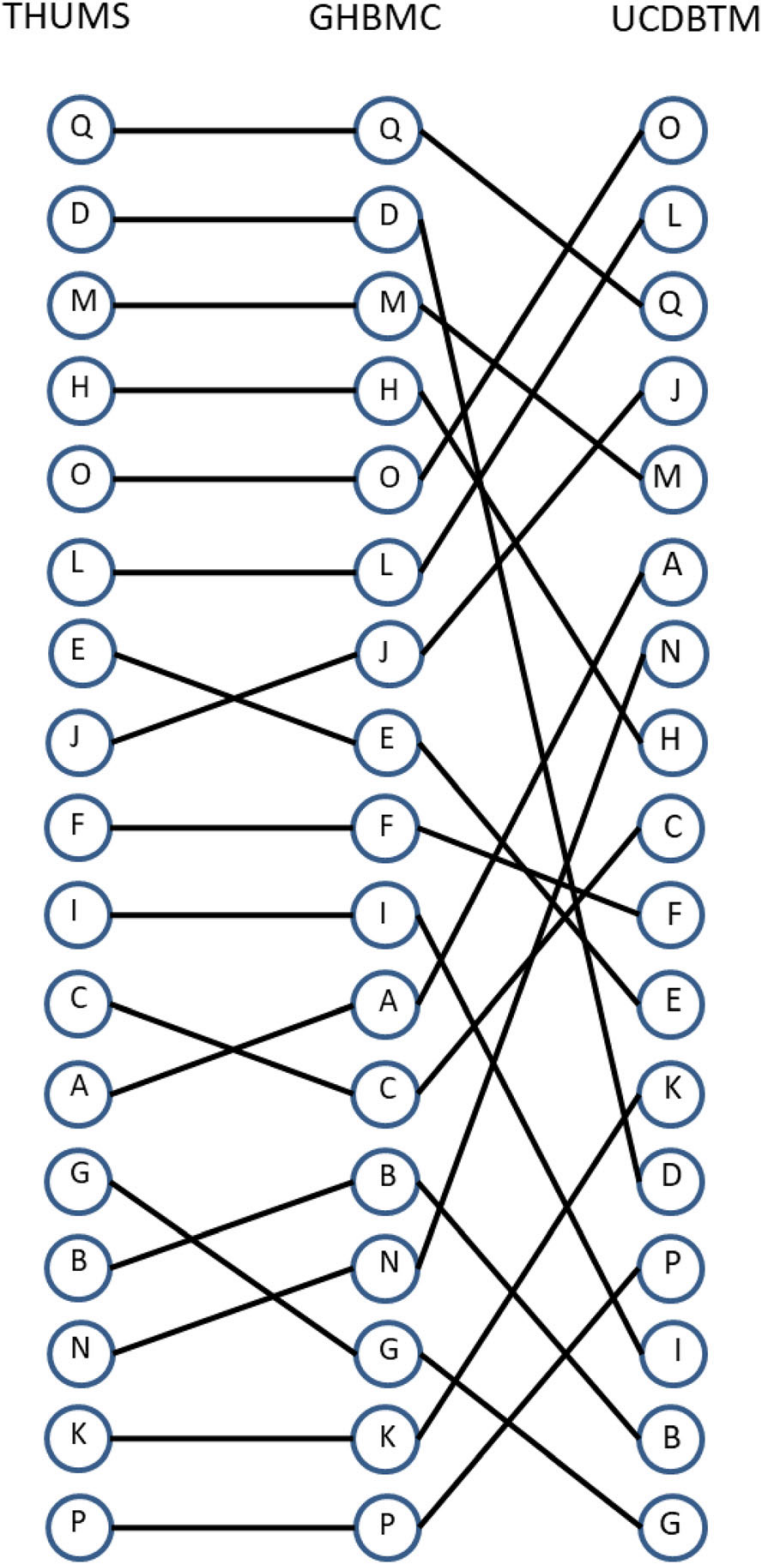
Visualization of the two models with the highest (left) and lowest (right) Kendall’s tau. GHBMC and THUMS had a Kendall’s tau of 0.95 and GHBMC and UCDBTM a Kendall’s tau of 0.51. The circles indicate the different helmets from A to Q with the best performing helmet at the top and the worst performing helmet at the bottom. The lines between the circles are pulled between the same helmet for the different brain models.

Kendall’s tau varied between 0.50 and 0.98 for the different model metrics when evaluating all loading conditions together (Table 3). The tau coefficient was relatively high between all brain models (>0.8) except for the UCDTBM, IC, and SIMon models. A visualization of the lowest and highest Kendall’s tau between the different model prediction of strain is shown in Figure 4. Kendall’s tau was often lower in the Zrot loading conditions compared to Xrot and Yrot (see further details in the supplementary materials). For the Zrot loading condition, the lowest coefficient of variation was also seen between the seventeen different helmets, 3–14% depending on the model, compared to 11–83% for Xrot and 11–46% for Yrot (see supplementary materials). The ranking from the worst to best performing helmet can also be found in the supplementary materials.

A Kendall’s tau larger than 0.8 was found for most of the brain models when compared to kinematic-based metrics based on angular velocity (Table 5). Only the IC model (strain) showed a Kendall’s tau above 0.8 for PAA. All brain models showed a Kendall’s tau below 0.62 when compared to PLA, HIC and STAR*.

**Table 5:** Kendall’s tau between the different models and the different kinematic-based metrics.

	PLA	HIC	PAA	PAV	BrIC	UBrIC	DAMAGE	STAR*
GHBMC	− 0.115	− 0.361	0.59	**0.893**	**0.935**	**0.904**	**0.945**	0.499
GHBMC CSDM	− 0.131	− 0.377	0.576	**0.895**	**0.942**	**0.911**	**0.953**	0.482
IC	0.084	− 0.14	**0.867**	0.655	0.636	0.627	0.631	0.616
IC strain rate	− 0.024	− 0.258	0.746	**0.804**	0.760	0.747	0.724	0.555
KTH	− 0.079	− 0.319	0.647	**0.868**	**0.868**	**0.840**	**0.876**	0.540
PIPER	− 0.098	− 0.335	0.650	**0.925**	**0.865**	**0.837**	**0.867**	0.534
SIMon	− 0.08	− 0.314	0.673	**0.852**	**0.802**	0.799	0.763	0.521
SIMon CSDM	− 0.095	− 0.332	0.650	**0.871**	**0.827**	**0.818**	**0.82**	0.515
THUMS	− 0.117	− 0.36	0.609	**0.903**	**0.912**	**0.884**	**0.945**	0.506
UCDBTM	0.076	− 0.156	0.712	0.535	0.504	0.494	0.494	0.521
WHIM	− 0.075	− 0.302	0.677	**0.885**	**0.845**	**0.823**	0.796	0.542

Highlighted in bold above 0.80

### Rating of Helmets

**Figure 5:**
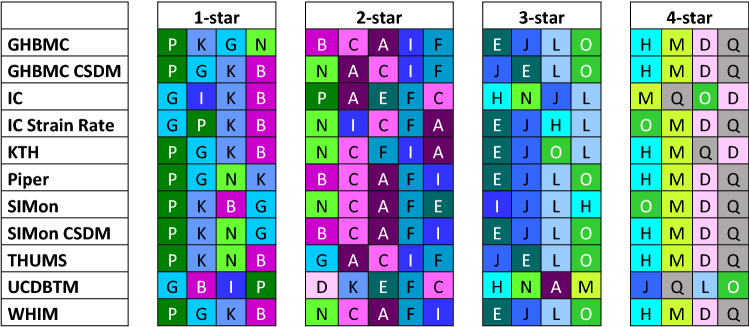
The rating of helmet based on different brain models. The different colors are indicating the different helmets.

In general, there was a reasonable agreement in how the helmets were rated using the different brain models. Eleven of the seventeen helmets were given the same rating by at least 10 of the 11 brain model metrics, and sixteen of seventeen helmets given the same rating by at least 7 of the 11 brain model metrics. All brain model metrics rated helmet M and Q as the best performing helmet when combining all three loading conditions (Figure 5). Likewise, helmets P, K, and G were rated in the bottom group (1-star) by 10 of the 11 brain models. Only helmets I and N were given 3 different ratings (1-, 2-, and 3-star). Helmet N had the most rating disparity of all the helmets (4 for 1-star, 5 for 2-star, and 2 for 3-star). Helmet D was rated 4-star by 10 of 11 brain models but was rated as a 2-star helmet by the UCDBTM.

**Figure 6:**
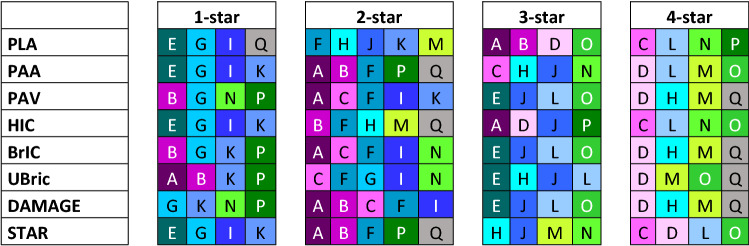
The rating of helmets based on different kinematic-based metrics. The different colors are indicating the different helmets.

All brain models showed the best correlation with the ranking of kinematic-based metrics based on rotation (Table 5). All rotation-based metrics rated helmet D as a 4-star helmet and PAV, BrIC, UBrIC, and DAMAGE all rated helmet M and Q as a 4-star helmet (Figure 6). Including PAA, these kinematic-based metrics assigned star ratings to each helmet, similar to what was assigned using the FE brain models.

## Discussion

This study shows how different FE brain models and kinematic-based metrics rank and rate a large number of bicycle helmets. Seventeen different helmets available on the Swedish market (2015) were ranked and rated based on three oblique impacts that produce rotations about the three anatomical axes of the head. The results from the eight different brain models, with multiple outputs from some models, and eight different kinematic-based metrics showed that the choice of metrics could influence the ranking and rating as well as the linear correlation. Comparing the ranking using Kendall’s tau showed a high correlation (above 0.8) for 30 of the 55 model-to-model comparisons (Table 3). Pearson’s *r*^2^ showed a correlation above 0.8 for 45 of the 55 model-to-model comparisons. Thus, there was generally a good correlation between different models using the bicycle helmet oblique impact dataset. It is important to note that the lower correlation between models is not necessarily a measure of the quality or accuracy of individual models. To be able to evaluate the quality and accuracy of the models further specification concerning quality measures is required as well as further validation, good experiments to validate the models against and an objective evaluation of the validation. In fact, it is an ongoing effort to understand how best to validate a model and rate its quality, as discussed recently.^26^,^54^,^56^

The rating of the helmets from 1- to 4-star were broadly similar. Some helmets had a difference in rating, depending on the choice of brain model. However, two helmets had larger differences. Helmets I and N were ranked with 1-star for some brain models, and either 2- or 3-star for some other brain models. For helmet N, most of the models placed it among the highest-ranked 1-star helmets or among the lowest-ranked 2-star helmets. The peak metrics for the different models were rather close, so differences in star rating were more or less dependent on the percentile boundary values that define each rating group with two exceptions: the UCDTBM and IC models, both rated the helmet as a 3-star helmet. The rating presented in Figure 5 was based on the average value of the three loading conditions. As can be seen in the supplementary materials, the difference for these two models compared to the other models was mainly due to the difference in the ranking of the helmets for the Xrot loading condition and especially for Zrot loading condition. For Yrot, the ranking position for helmet N was almost the same for all models. For helmet I, the difference was mainly for the Zrot loading conditions. Zrot was also the direction that had the smallest coefficient of variation, which could influence the fact that lower correlation was seen for this loading condition. Also, helmet D had some more variation in rating, particularly with the UCDTBM. The UCDTBM ranked the helmet with 2-star, whereas the other brain models ranked the helmet as a 4-star helmet. The ranking of the UCDTBM differed mostly for the Xrot loading condition (10–13 positions difference) compared to Yrot (2–4 positions) and Zrot (6–7 positions).

It is not clear what factors related to the construction of one brain model determine its difference from the others. IC, KTH, PIPER and WHIM use the same material model and model parameters derived by Kleiven,^33^ which account for tension-compression asymmetry. Still, the linear correlation and correlation of ranking were not always highest between these models. Other models such as GHBMC, SIMon, THUMS, and UCDTBM have used a linear viscoelastic material model for the brain tissue, which does not capture the full nonlinear response observed in some tissue studies.^17^,^39^ However, differences in the material models and properties do not seen to be a major factor affecting the correlation of model responses and the correlation of ranking. For instance, the KTH (which uses a nonlinear material model for the brain) and GHBMC (which uses a linear model for the brain) show high correlation in ranking compared to models with either linear or nonlinear material models. When comparing the UCDTBM to the other models in the context of the number of elements and nodes, brain volume, material properties, etc. there is no significant difference compared to other models. The UCDTBM is in the medium range of the selection of models when it comes to the number of nodes and elements. Abaqus is used to solve the UCDTBM, nonetheless, the same software is also used for WHIM. It should be mentioned that the IC and UCDTBM models were the only models that showed a higher correlation to the PAA compared to PAV, which could make a difference in ranking.

Besides material properties, brain element mesh density, mesh quality, element formulation, solver, and hourglass control could all significantly affect strain predictions. Earlier studies have shown models with finer mesh would lead to large brain strains when other modeling parameter are the same.^27^,^53^ Similarly, the variations in mesh density among the eight brain models may contribute to the difference in strain predictions. However, it is difficult to isolate and quantify the effect that the differences in numerical approaches has on the correlations between models and their rankings, because these factors are often interactive. Future studies may investigate the interactive effects of key modelling choices on predictions of the human head FE models, as a step towards providing guidance on using such models for ranking head protection systems.

IC, GHBMC, and SIMon models were evaluated for different local metrics since these various metrics have been used to evaluate the effect on brain tissue in previous studies.^19^,^45^ For both GHBMC and SIMon models, MPS and CSDM were evaluated, and small differences were seen in the correlation between these two metrics (Kendall’s tau of 0.98 and 0.94, respectively). A slightly larger difference was seen for the IC model when evaluating strain and strain rate with Kendall’s tau of 0.84. These differences in the ranking only had a small influence on the helmet rating, which most often is the only information that is provided to consumers. For the GHBMC model, two helmets had different star rating (Helmet B and N), and for IC and SIMon models, four helmets had different star rating (Helmet E, I, N, and P for IC model, and E, H, I, O for SIMon model).

This study focused on the helmet ranking and rating using different brain models rather than evaluating the biofidelity of the models, e.g., through comparing their predictions with data from experiments on post-mortem human subjects (PMHS) or human volunteers. Most of the models have been evaluated against different PMHS experiments, and in some cases also against volunteer data (see the Supplementary material Table S3). However, it is difficult to compare the different validation results between the models due to differences in the validation process. There are some exceptions, e.g., Giordano & Kleiven^26^ evaluated the THUMS, isotropic KTH, and GHBMC models with the same methodology. In total, 40 experiments were evaluated. They found a biofidelity rating derived from correlation and analysis (CORA) score between 5.80 and 6.23, which was indicative of fair biofidelity for all three models. The ranking in this study showed that Kendall’s tau varied between 0.90 and 0.95 and only with a minor difference in helmet rating between these models, but this is not necessarily due to the fact that they have similar correlation against PMHS. Trotta *et al.*^50^ used the same evaluation protocol for one set of experiments with the UCDBTM and found higher scores compared to the GHBMC, and THUMS models, but when comparing the correlation of ranking to other models, UCDBTM showed the lowest values. Nevertheless, a recent study by Zhao and Ji^54^ suggests that CORA may not be effective in discriminating brain injury models in terms of whole-brain strain, after all, as two models with similar CORA scores could produce whole-brain strains 2–3 times difference in magnitude.

The ranking of helmets was also evaluated using some kinematic-based metrics. The rating of the helmets was vastly different for the metrics based on linear acceleration compared with the metrics predicted by the brain models. In terms of PLA, helmet Q was rated as a 1-star helmet, and all brain models rated that helmet as a 4- star helmet. PLA and HIC have shown better correlation to skull fracture than brain injuries.^23^,^34^ In this study, all brain models apply the dummy headform kinematics through a rigid skull, and only the response of the brain tissue is included in the comparison. In this sense, the models are only able to assess the risk of diffuse-type brain injuries (e.g., concussion, diffuse axonal injury), which arise primarily from brain deformation mechanisms^22^,^40^ resulting from head rotation. For future test standards and rating methods, it may be necessary to evaluate both the risk of skull fracture and a broader spectrum of brain injuries.

The kinematic-based metrics based on the angular motion had the highest correlation with the metrics predicted by the brain models, but metrics with the highest correlation were dependent on the model used. In some cases, the different metrics had a rather similar correlation coefficient both for ranking, and linear correlation. For most models, PAA showed a lower correlation compared to the other angular metrics with the exception of the IC model with strain and the UCDTBM model. Some previous studies^21^,^30^,^32^,^48^ have proposed that angular velocity shows a better correlation to brain responses for short duration pulses (10–20 ms), that are characteristic of helmet pulses, while angular acceleration plays a larger role for pulses with longer durations, e.g., automotive collisions. These present results are only for short duration helmet impact pulses.

Different rating methods have been presented previously, e.g., Deck *et al.*^11^ and Stigson.^46^ Those two studies were based on brain model response, but they rated the helmets based on the injury risk functions. In the present study, we rated the helmets based on the MPS/strain rate or CSDM directly without any assessment against injury risk curves. There were two reasons for this. Firstly, not all brain models used in this study have had injury risk functions developed specifically for them. Secondly, the data and methods for developing injury risk functions are changing rapidly with ongoing research. In the literature, some model developers use different types of brain injuries to create AIS2+ risk curves based on simulations of various accident situations.^42^ Others have developed the risk functions from reconstructions of concussion cases,^5^,^33^ a combination of football reconstructions and volunteer sled test data^43^,^44^ or scaled animal data.^49^ In future, it would also be wise to explore in more detail what is required from these injury risk functions and the underlying cases on which the risk functions are based. Whether and how risk functions should be used ought to be discussed in the research community, in addition to what particular inclusion criteria should be used when choosing the uninjured and injured populations. Ideally, this should be available to the scientific research community through an open-access database.

Since no injury risk functions were used when rating the helmets, the rating should not be interpreted as the absolute real-world performance of the helmet, rather the performance of that helmet with the conditions imposed to that particular brain model using that injury metric. As mentioned above, in the present study, the helmet rating was based on the mean value of three different impact conditions, and the star ratings were distributed depending on the 25th, 50th, and 75th percentile values of the seventeen different helmets. With this system the helmets are forced into four different categories. It could be so that all helmet had a low risk of injury and should be rated high or had a high risk of injury and should be rated low, but now the helmets are even distributed over the four categories of stars. For Zrot the coefficient of variation between the helmets was relatively small, the mean values of the metrics used to determine the boundaries of the ratings were also relatively close (see supplementary materials). If injury risk functions would have been used, it is possible that all the helmets would have been ranked in only one or two categories. With the system used in this study, in some cases, the response of two helmets was similar in their performance, but they were rated differently because their performance lay on opposite sides of the border between two rating categories. This would, of course, influence Kendall’s tau, and the rating methods.

Another proposed rating method for bicycle helmets is the STAR rating by Virginia Tech.^4^ They use a combination of peak linear acceleration and peak change in angular velocity for six impact locations using two impact velocities to calculate the STAR value. Since this study only included one impact velocity and three different impact locations, a modified STAR value (STAR*) was used. STAR* shows a lower correlation against the models both for linear correlation and ranking compared to the kinematic-based metrics that only depended on angular motion.

This study included eight different brain models, which to the authors’ knowledge is the most extensive comparison study to date. However, other brain models do exist and are used to assess safety products, although they have not been included in this study. In addition, there are also other metrics based on global kinematics that were not evaluated in this study. For the brain models, the effect on brain tissue has only been evaluated on whole-brain level, but there are studies suggesting that metrics on subregion levels would be a better predictor of brain injuries (e.g., Wu *et al.*^52^).

This study is based on experiments of bicycle helmet impacts that were performed without the neck and the rest of the body. It is unclear how important the neck and body are. Previous studies have shown divergent results when it comes to headfirst helmet impacts.^14^,^16^,^25^ The results have been influenced by the impact conditions such as impact point and impact surface. By including the neck and body, a biphasic acceleration with an acceleration phase and a deceleration phase could, which could amplify the brain strain.^38^,^55^ The deceleration phase is not included in the current study since all tests are performed without the neck. This is a limitation of the current study and different helmet rating programs,^4^,^11^,^47^ which may be addressed in future by the development of a neck that is more biofidelic in head first impacts.

From the models and kinematic-based metrics included in this study, the results show that the ranking and rating can be influenced by the choice of the assessment metric. There is a potential risk if different rating methods present different results depending on which FE model or kinematic-based metric inform their rating method. This is likely to cause confusion among consumers rather than provide constructive advice regarding the relative safety performance of helmets. Rating methods are best used to allow consumers to distinguish between a safer and less safe helmets, whereas test standards are intended to exclude unsafe helmets from the marketplace. We strongly suggest that the biomechanics community work collaboratively to reach consensus on a validation procedure for FE models of the head. This procedure should involve both validation against experimental data and comparison to real life accident so that derive trustworthy injury risk functions. However, as discussed above, we do not recommend at present that injury risk curves be used in helmet rating methods because the data and methods for developing injury risk functions are changing rapidly with ongoing research.

Nevertheless, in order to provide specific recommendations, further knowledge and technology developments are necessary. For example, more data based on real-world accidents are required to evaluate the performance of the injury metrics. A consensus on a standardized procedure to validate brain injury models and rate the performance is also needed to establish the confidence in their practical applications. In addition, injury risk functions based on real bicycle accidents with injured and non-injured casesare also needed for applications specific to bicycle helmets. At present, depending on which model or injury metric that is chosen to evaluate the helmet performance, the ranking and rating can differ. We suggest that all rating organizations should provide clear information regarding the uncertainty in the rating depending on the metric used.

## Electronic supplementary material

Below is the link to the electronic supplementary material.

Electronic supplementary material 1 (DOCX 2311 kb)
