# Supplementary material for: Ranking and Rating Bicycle Helmet Safety Performance in Oblique Impacts Using Eight Different Brain Injury Models
Source: Ann Biomed Eng. 2021 Jan 21;49(3):1097–109. doi: 10.1007/s10439-020-02703-w (PMC7952345; doi:10.1007/s10439-020-02703-w)
Supplement: Supplementary file 1 — Electronic supplementary material 1 (DOCX 2311 kb) [file 10439_2020_2703_MOESM1_ESM.docx]

# Supplementary Materials

The kinematics from the experiments are presented in Figure S1. A decrease of the angular velocity can be seen for the Zrot impacts. The decrease of the angular velocity in this loading conditions is most probably due to the fact that the rear part of the helmet hits the anvil, and the interaction between the foam and the headform. When the helmet impacts the anvil, some roll of the helmet at the anvil can be seen. The foam is also compressed and then there is a possible spring-back, which is causing the reduction of angular velocity.


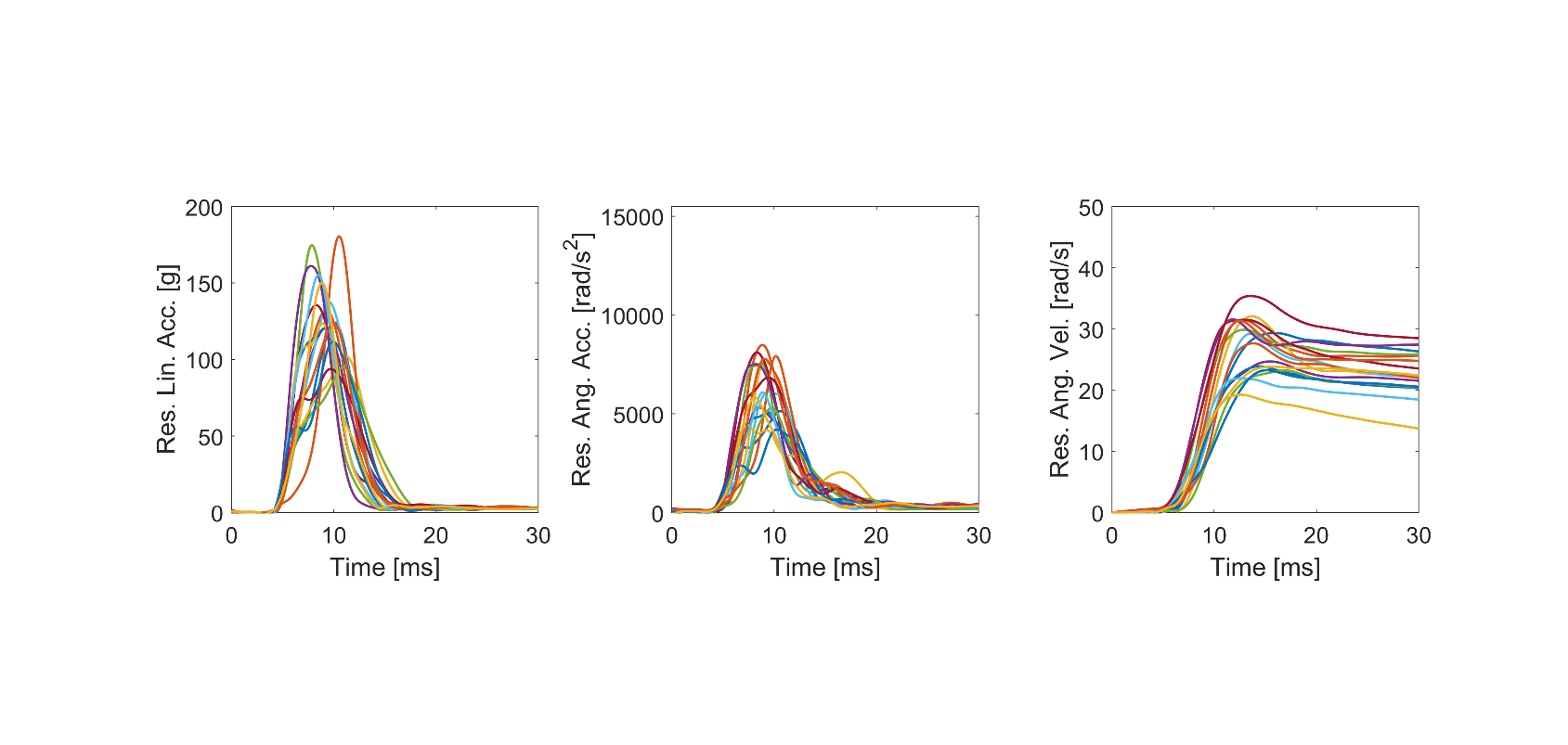


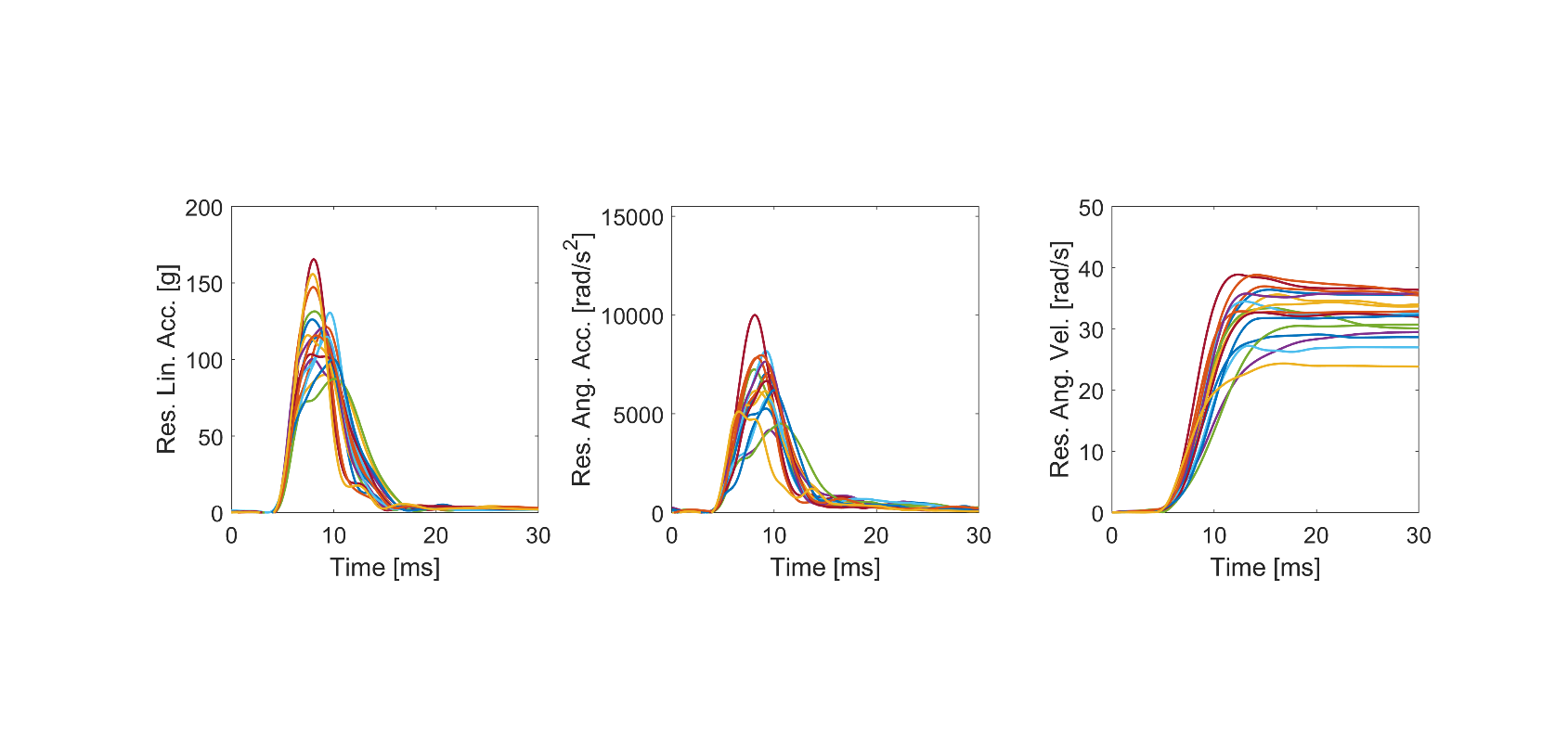


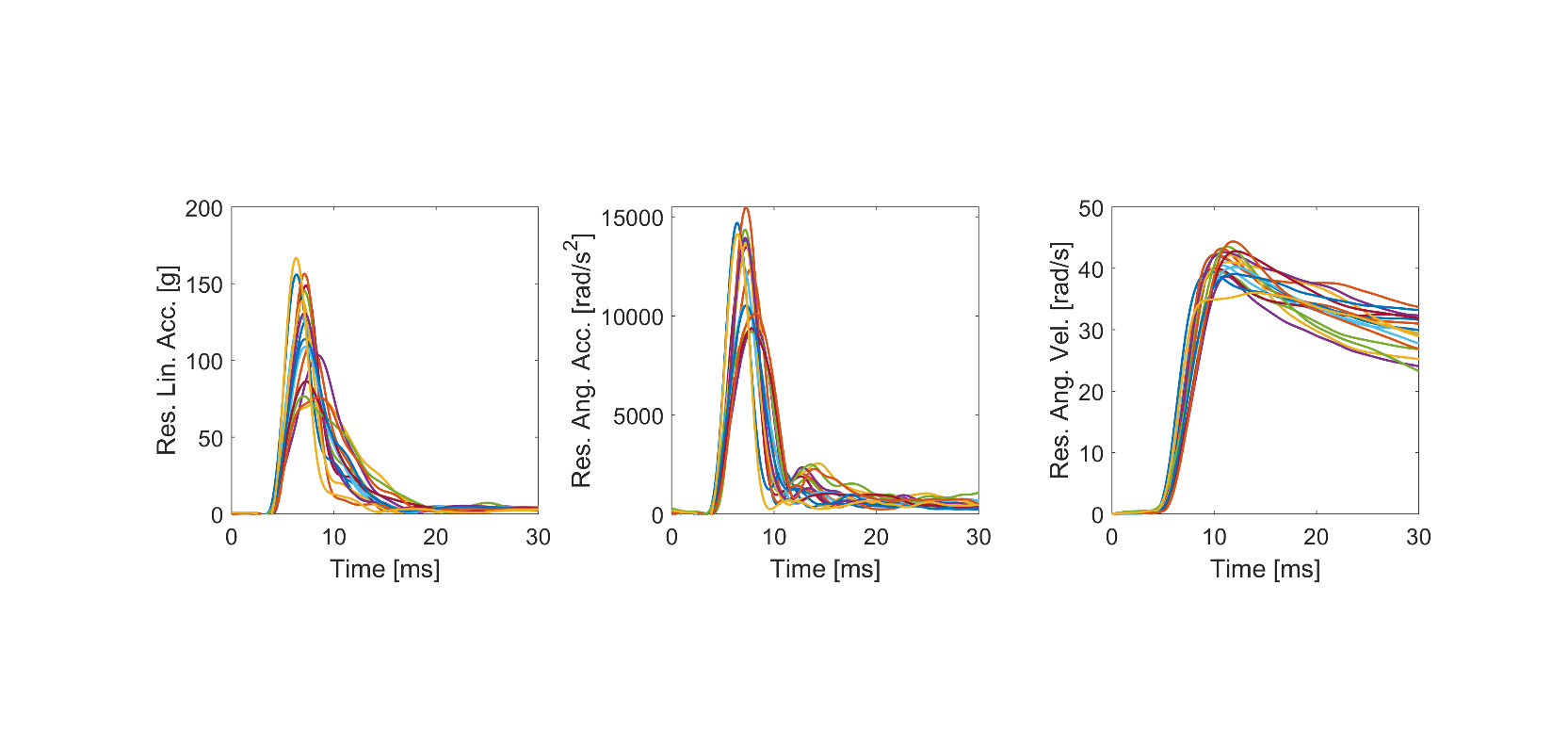


**Figure S1.** The kinematics from all tests, from left to right linear acceleration, angular acceleration, and angular velocity, from top to bottom Xrot, Yrot, and Zrot.

| GHBMC  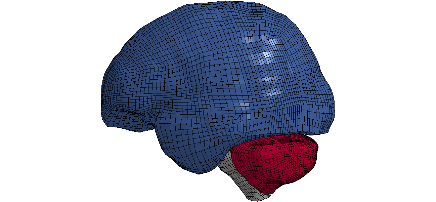 | IC  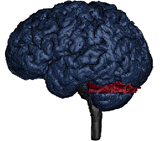 | KTH  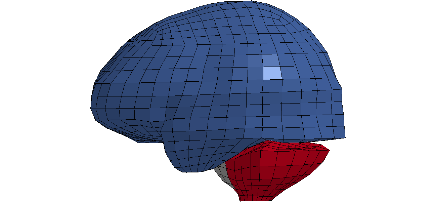 |
| --- | --- | --- |
| PIPER  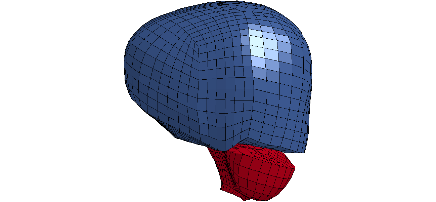 | SIMon  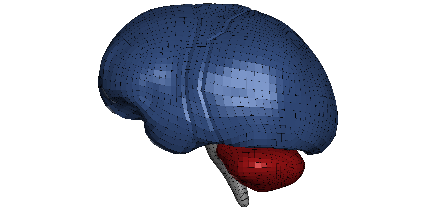 | THUMS  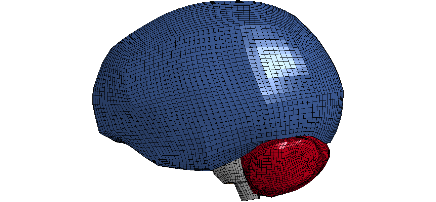 |
| UCDTBM  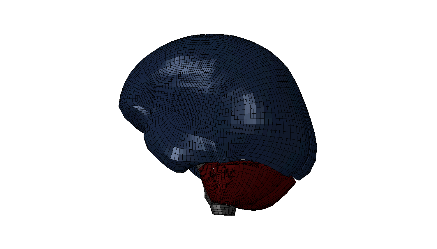 | WHIM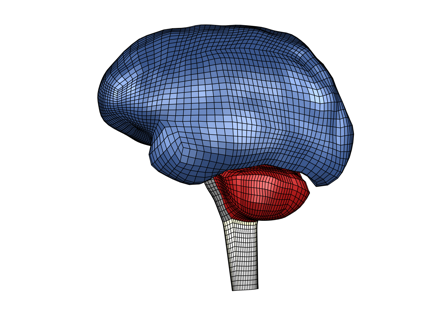 |  |

**Figure S2.** The geometry and mesh of the different brain models.

**Table S1.** Kendall's tau per loading condition for the different brain models.

| **GHBMC** | **Xrot** | **Yrot** | **Zrot** | **GHBMC CSDM** | **Xrot** | **Yrot** | **Zrot** | **IC** | **Xrot** | **Yrot** | **Zrot** |
| --- | --- | --- | --- | --- | --- | --- | --- | --- | --- | --- | --- |
| **GHBMC** | **-** | **-** | **-** | **GHBMC** | **0.96** | **0.99** | **0.92** | **GHBMC** | 0.66 | 0.69 | 0.27 |
| **GHBMC CSDM** | **0.96** | **0.99** | **0.92** | **GHBMC CSDM** | **-** | **-** | **-** | **GHBMC CSDM** | 0.62 | 0.71 | 0.24 |
| **IC** | 0.66 | 0.69 | 0.27 | **IC** | 0.62 | 0.71 | 0.24 | **IC** | **-** | **-** | **-** |
| **IC Strain Rate** | **0.82** | 0.79 | 0.70 | **IC Strain Rate** | **0.84** | **0.81** | 0.63 | **IC Strain Rate** | 0.60 | **0.90** | 0.54 |
| **KTH** | 0.77 | **0.88** | 0.79 | **KTH** | 0.75 | **0.87** | 0.72 | **KTH** | 0.72 | **0.81** | 0.49 |
| **PIPER** | **0.82** | **0.91** | **0.95** | **PIPER** | **0.87** | **0.93** | **0.91** | **PIPER** | 0.54 | 0.78 | 0.29 |
| **SIMon** | **0.84** | **0.91** | 0.72 | **SIMon** | **0.85** | **0.93** | 0.75 | **SIMon** | 0.59 | 0.75 | 0.22 |
| **SIMon CSDM** | 0.46 | **0.96** | **0.82** | **SIMon CSDM** | 0.50 | **0.97** | **0.85** | **SIMon CSDM** | 0.47 | 0.71 | 0.18 |
| **THUMS** | **0.81** | **0.99** | **0.85** | **THUMS** | **0.85** | **0.97** | 0.77 | **THUMS** | 0.56 | 0.71 | 0.27 |
| **UCDBTM** | 0.54 | 0.34 | 0.11 | **UCDBTM** | 0.52 | 0.36 | 0.03 | **UCDBTM** | 0.54 | 0.53 | 0.59 |
| **WHIM** | **0.85** | **0.87** | 0.79 | **WHIM** | **0.90** | **0.88** | 0.77 | **WHIM** | 0.57 | **0.82** | 0.35 |
| **IC Strain Rate** | **Xrot** | **Yrot** | **Zrot** | **KTH** | **Xrot** | **Yrot** | **Zrot** | **PIPER** | **Xrot** | **Yrot** | **Zrot** |
| **GHBMC** | **0.82** | 0.79 | 0.70 | **GHBMC** | 0.77 | **0.88** | 0.79 | **GHBMC** | **0.82** | **0.91** | **0.95** |
| **GHBMC CSDM** | **0.84** | **0.81** | 0.63 | **GHBMC CSDM** | 0.75 | **0.87** | 0.72 | **GHBMC CSDM** | **0.87** | **0.93** | **0.91** |
| **IC** | 0.60 | **0.90** | 0.54 | **IC** | 0.72 | **0.81** | 0.49 | **IC** | 0.54 | 0.78 | 0.29 |
| **IC Strain Rate** | **-** | **-** | **-** | **IC Strain Rate** | 0.74 | **0.91** | 0.77 | **IC Strain Rate** | **0.85** | **0.88** | 0.69 |
| **KTH** | 0.74 | **0.91** | 0.77 | **KTH** | **-** | **-** | **-** | **KTH** | 0.71 | **0.91** | **0.81** |
| **PIPER** | **0.85** | **0.88** | 0.69 | **PIPER** | 0.71 | **0.91** | **0.81** | **PIPER** | **-** | **-** | **-** |
| **SIMon** | **0.96** | **0.85** | 0.59 | **SIMon** | 0.75 | **0.88** | 0.56 | **SIMon** | **0.87** | **0.97** | 0.66 |
| **SIMon CSDM** | 0.49 | **0.81** | 0.52 | **SIMon CSDM** | 0.40 | **0.87** | 0.60 | **SIMon CSDM** | 0.57 | **0.93** | 0.77 |
| **THUMS** | **0.87** | **0.81** | 0.54 | **THUMS** | 0.72 | **0.90** | 0.69 | **THUMS** | **0.99** | **0.93** | 0.79 |
| **UCDBTM** | 0.57 | 0.49 | 0.33 | **UCDBTM** | 0.63 | 0.40 | 0.28 | **UCDBTM** | 0.57 | 0.40 | 0.09 |
| **WHIM** | **0.94** | **0.93** | **0.81** | **WHIM** | 0.71 | **0.93** | 0.72 | **WHIM** | **0.91** | **0.96** | 0.77 |
| **SIMon** | **Xrot** | **Yrot** | **Zrot** | **SIMon-CSDM** | **Xrot** | **Yrot** | **Zrot** | **THUMS** | **Xrot** | **Yrot** | **Zrot** |
| **GHBMC** | **0.84** | **0.91** | 0.72 | **GHBMC** | 0.46 | **0.96** | **0.82** | **GHBMC** | **0.81** | **0.99** | **0.85** |
| **GHBMC CSDM** | **0.85** | **0.93** | 0.75 | **GHBMC CSDM** | 0.50 | **0.97** | **0.85** | **GHBMC CSDM** | **0.85** | **0.97** | 0.77 |
| **IC** | 0.59 | 0.75 | 0.22 | **IC** | 0.47 | 0.71 | 0.18 | **IC** | 0.56 | 0.71 | 0.27 |
| **IC Strain Rate** | **0.96** | **0.85** | 0.59 | **IC Strain Rate** | 0.49 | **0.81** | 0.52 | **IC Strain Rate** | **0.87** | **0.81** | 0.54 |
| **KTH** | 0.75 | **0.88** | 0.56 | **KTH** | 0.40 | **0.87** | 0.60 | **KTH** | 0.72 | **0.90** | 0.69 |
| **PIPER** | **0.87** | **0.97** | 0.66 | **PIPER** | 0.57 | **0.93** | 0.77 | **PIPER** | **0.99** | **0.93** | 0.79 |
| **SIMon** | **-** | **-** | **-** | **SIMon** | 0.50 | **0.96** | **0.84** | **SIMon** | **0.88** | **0.93** | 0.69 |
| **SIMon CSDM** | 0.50 | **0.96** | **0.84** | **SIMon CSDM** | **-** | **-** | **-** | **SIMon CSDM** | 0.56 | **0.97** | **0.85** |
| **THUMS** | **0.88** | **0.93** | 0.69 | **THUMS** | 0.56 | **0.97** | **0.85** | **THUMS** | **-** | **-** | **-** |
| **UCDBTM** | 0.58 | 0.40 | 0.06 | **UCDBTM** | 0.24 | 0.36 | 0.00 | **UCDBTM** | 0.58 | 0.36 | 0.12 |
| **WHIM** | **0.96** | **0.93** | 0.75 | **WHIM** | 0.54 | **0.88** | 0.68 | **WHIM** | **0.90** | **0.88** | 0.65 |

| **UCDBTM** | **Xrot** | **Yrot** | **Zrot** | **WHIM** | **Xrot** | **Yrot** | **Zrot** |  |  |  |  |
| --- | --- | --- | --- | --- | --- | --- | --- | --- | --- | --- | --- |
| **GHBMC** | 0.54 | 0.34 | 0.11 | **GHBMC** | **0.85** | **0.87** | 0.79 |  |  |  |  |
| **GHBMC CSDM** | 0.52 | 0.36 | 0.03 | **GHBMC CSDM** | **0.90** | **0.88** | 0.77 |  |  |  |  |
| **IC** | 0.54 | 0.53 | 0.59 | **IC** | 0.57 | **0.82** | 0.35 |  |  |  |  |
| **IC Strain Rate** | 0.57 | 0.49 | 0.33 | **IC Strain Rate** | **0.94** | **0.93** | **0.81** |  |  |  |  |
| **KTH** | 0.63 | 0.40 | 0.28 | **KTH** | 0.71 | **0.93** | 0.72 |  |  |  |  |
| **PIPER** | 0.57 | 0.40 | 0.09 | **PIPER** | **0.91** | **0.96** | 0.77 |  |  |  |  |
| **SIMon** | 0.58 | 0.40 | 0.06 | **SIMon** | **0.96** | **0.93** | 0.75 |  |  |  |  |
| **SIMon CSDM** | 0.24 | 0.36 | 0.00 | **SIMon CSDM** | 0.54 | **0.88** | 0.68 |  |  |  |  |
| **THUMS** | 0.58 | 0.36 | 0.12 | **THUMS** | **0.90** | **0.88** | 0.65 |  |  |  |  |
| **UCDBTM** | **-** | **-** | **-** | **UCDBTM** | 0.54 | 0.42 | 0.16 |  |  |  |  |
| **WHIM** | 0.54 | 0.42 | 0.16 | **WHIM** | **-** | **-** | **-** |  |  |  |  |

*Highlighted in bold above 0.80*

**Table S2.** Pearson's correlation factor r^2^ per loading condition.

| **GHBMC** | **Xrot** | **Yrot** | **Zrot** | **GHBMC CSDM** | **Xrot** | **Yrot** | **Zrot** | **IC** | **Xrot** | **Yrot** | **Zrot** |
| --- | --- | --- | --- | --- | --- | --- | --- | --- | --- | --- | --- |
| **GHBMC** | **-** | **-** | **-** | **GHBMC** | **0.97** | **0.98** | **0.99** | **GHBMC** | 0.77 | 0.73 | 0.33 |
| **GHBMC CSDM** | **0.97** | **0.98** | **0.99** | **GHBMC CSDM** | **-** | **-** | **-** | **GHBMC CSDM** | 0.69 | 0.77 | 0.29 |
| **IC** | 0.77 | 0.73 | 0.33 | **IC** | 0.69 | 0.77 | 0.29 | **IC** | **-** | **-** | **-** |
| **IC Strain Rate** | **0.91** | **0.89** | 0.79 | **IC Strain Rate** | **0.87** | **0.90** | 0.76 | **IC Strain Rate** | **0.88** | **0.91** | 0.68 |
| **KTH** | **0.90** | **0.95** | **0.82** | **KTH** | **0.82** | **0.94** | 0.78 | **KTH** | **0.89** | **0.86** | 0.53 |
| **PIPER** | **0.87** | **0.97** | **0.99** | **PIPER** | **0.86** | **0.96** | **0.98** | **PIPER** | 0.71 | 0.79 | 0.33 |
| **SIMon** | **0.93** | **0.97** | **0.91** | **SIMon** | **0.91** | **0.97** | **0.92** | **SIMon** | **0.84** | 0.79 | 0.22 |
| **SIMon CSDM** | **0.96** | **0.97** | **0.95** | **SIMon CSDM** | **0.97** | **0.99** | **0.97** | **SIMon CSDM** | 0.77 | 0.80 | 0.21 |
| **THUMS** | **0.94** | **0.99** | **0.97** | **THUMS** | **0.92** | **0.99** | **0.96** | **THUMS** | 0.77 | 0.76 | 0.29 |
| **UCDBTM** | 0.47 | 0.25 | 0.04 | **UCDBTM** | 0.44 | 0.24 | 0.02 | **UCDBTM** | 0.60 | 0.46 | 0.63 |
| **WHIM** | **0.93** | **0.94** | **0.89** | **WHIM** | **0.90** | **0.93** | **0.89** | **WHIM** | 0.80 | **0.81** | 0.33 |
| **IC Strain Rate** | **Xrot** | **Yrot** | **Zrot** | **KTH** | **Xrot** | **Yrot** | **Zrot** | **PIPER** | **Xrot** | **Yrot** | **Zrot** |
| **GHBMC** | **0.91** | **0.89** | 0.79 | **GHBMC** | **0.90** | **0.95** | **0.82** | **GHBMC** | **0.87** | **0.97** | **0.99** |
| **GHBMC CSDM** | **0.87** | **0.90** | 0.76 | **GHBMC CSDM** | **0.82** | **0.94** | 0.78 | **GHBMC CSDM** | **0.86** | **0.96** | **0.98** |
| **IC** | **0.88** | **0.91** | 0.68 | **IC** | **0.89** | **0.86** | 0.53 | **IC** | 0.71 | 0.79 | 0.33 |
| **IC Strain Rate** | **-** | **-** | **-** | **IC Strain Rate** | **0.89** | **0.97** | 0.77 | **IC Strain Rate** | **0.94** | **0.96** | 0.79 |
| **KTH** | **0.89** | **0.97** | 0.77 | **KTH** | **-** | **-** | **-** | **KTH** | 0.76 | **0.98** | **0.85** |
| **PIPER** | **0.94** | **0.96** | 0.79 | **PIPER** | 0.76 | **0.98** | **0.85** | **PIPER** | **-** | **-** | **-** |
| **SIMon** | **0.98** | **0.95** | 0.72 | **SIMon** | **0.86** | **0.97** | 0.58 | **SIMon** | **0.93** | **1.00** | **0.87** |
| **SIMon CSDM** | **0.92** | **0.91** | 0.67 | **SIMon CSDM** | **0.84** | **0.95** | 0.65 | **SIMon CSDM** | **0.89** | **0.96** | **0.92** |
| **THUMS** | **0.96** | **0.92** | 0.69 | **THUMS** | **0.84** | **0.96** | 0.80 | **THUMS** | **0.98** | **0.99** | **0.94** |
| **UCDBTM** | 0.57 | 0.40 | 0.27 | **UCDBTM** | 0.54 | 0.32 | 0.14 | **UCDBTM** | 0.52 | 0.30 | 0.03 |
| **WHIM** | **0.98** | **0.98** | **0.87** | **WHIM** | **0.83** | **0.97** | 0.68 | **WHIM** | **0.97** | **0.99** | **0.90** |
| **SIMon** | **Xrot** | **Yrot** | **Zrot** | **SIMon-CSDM** | **Xrot** | **Yrot** | **Zrot** | **THUMS** | **Xrot** | **Yrot** | **Zrot** |
| **GHBMC** | **0.93** | **0.97** | **0.91** | **GHBMC** | **0.96** | **0.97** | **0.95** | **GHBMC** | **0.94** | **0.99** | **0.97** |
| **GHBMC CSDM** | **0.91** | **0.97** | **0.92** | **GHBMC CSDM** | **0.97** | **0.99** | **0.97** | **GHBMC CSDM** | **0.92** | **0.99** | **0.96** |
| **IC** | **0.84** | 0.79 | 0.22 | **IC** | 0.77 | 0.80 | 0.21 | **IC** | 0.77 | 0.76 | 0.29 |
| **IC Strain Rate** | **0.98** | **0.95** | 0.72 | **IC Strain Rate** | **0.92** | **0.91** | 0.67 | **IC Strain Rate** | **0.96** | **0.92** | 0.69 |
| **KTH** | **0.86** | **0.97** | 0.58 | **KTH** | **0.84** | **0.95** | 0.65 | **KTH** | **0.84** | **0.96** | 0.80 |
| **PIPER** | **0.93** | **1.00** | **0.87** | **PIPER** | **0.89** | **0.96** | **0.92** | **PIPER** | **0.98** | **0.99** | **0.94** |
| **SIMon** | **-** | **-** | **-** | **SIMon** | **0.97** | **0.97** | **0.96** | **SIMon** | **0.96** | **0.99** | **0.88** |
| **SIMon CSDM** | **0.97** | **0.97** | **0.96** | **SIMon CSDM** | **-** | **-** | **-** | **SIMon CSDM** | **0.94** | **0.98** | **0.95** |
| **THUMS** | **0.96** | **0.99** | **0.88** | **THUMS** | **0.94** | **0.98** | **0.95** | **THUMS** | **-** | **-** | **-** |
| **UCDBTM** | 0.53 | 0.30 | 0.02 | **UCDBTM** | 0.47 | 0.26 | 0.00 | **UCDBTM** | 0.54 | 0.26 | 0.02 |
| **WHIM** | **0.98** | **0.99** | **0.91** | **WHIM** | **0.95** | **0.94** | **0.84** | **WHIM** | **0.98** | **0.96** | 0.78 |

| **UCDBTM** | **Xrot** | **Yrot** | **Zrot** | **WHIM** | **Xrot** | **Yrot** | **Zrot** |  |  |  |  |
| --- | --- | --- | --- | --- | --- | --- | --- | --- | --- | --- | --- |
| **GHBMC** | 0.47 | 0.25 | 0.04 | **GHBMC** | **0.93** | **0.94** | **0.89** |  |  |  |  |
| **GHBMC CSDM** | 0.44 | 0.24 | 0.02 | **GHBMC CSDM** | **0.90** | **0.93** | **0.89** |  |  |  |  |
| **IC** | 0.60 | 0.46 | 0.63 | **IC** | 0.80 | **0.81** | 0.33 |  |  |  |  |
| **IC Strain Rate** | 0.57 | 0.40 | 0.27 | **IC Strain Rate** | **0.98** | **0.98** | **0.87** |  |  |  |  |
| **KTH** | 0.54 | 0.32 | 0.14 | **KTH** | **0.83** | **0.97** | 0.68 |  |  |  |  |
| **PIPER** | 0.52 | 0.30 | 0.03 | **PIPER** | **0.97** | **0.99** | **0.90** |  |  |  |  |
| **SIMon** | 0.53 | 0.30 | 0.02 | **SIMon** | **0.98** | **0.99** | **0.91** |  |  |  |  |
| **SIMon CSDM** | 0.47 | 0.26 | 0.00 | **SIMon CSDM** | **0.95** | **0.94** | **0.84** |  |  |  |  |
| **THUMS** | 0.54 | 0.26 | 0.02 | **THUMS** | **0.98** | **0.96** | 0.78 |  |  |  |  |
| **UCDBTM** | **-** | **-** | **-** | **UCDBTM** | 0.53 | 0.33 | 0.07 |  |  |  |  |
| **WHIM** | 0.53 | 0.33 | 0.07 | **WHIM** | **-** | **-** | **-** |  |  |  |  |

*Highlighted in bold above 0.80*

|  |  |  |  |  |  |  |  |  |  |  |  |  |  |  |  |  |  | **Average** | **SD** | **CV** |
| --- | --- | --- | --- | --- | --- | --- | --- | --- | --- | --- | --- | --- | --- | --- | --- | --- | --- | --- | --- | --- |
| **GHBMC** | N | K | G | C | B | P | E | A | I | F | Q | H | D | J | L | M | O |  |  |  |
|  | 0.285 | 0.257 | 0.256 | 0.250 | 0.247 | 0.240 | 0.231 | 0.225 | 0.222 | 0.219 | 0.204 | 0.199 | 0.193 | 0.191 | 0.188 | 0.187 | 0.186 | 0.222 | 0.029 | 13% |
| **GHBMC CSDM** | N | K | G | C | B | P | E | A | F | I | H | Q | D | J | L | O | M |  |  |  |
|  | 0.094 | 0.058 | 0.057 | 0.053 | 0.049 | 0.040 | 0.035 | 0.028 | 0.026 | 0.025 | 0.011 | 0.010 | 0.008 | 0.006 | 0.005 | 0.004 | 0.003 | 0.030 | 0.025 | 83% |
| **IC** | B | G | P | K | C | N | E | I | F | Q | H | M | L | D | A | J | O |  |  |  |
|  | 0.136 | 0.131 | 0.128 | 0.127 | 0.125 | 0.120 | 0.113 | 0.104 | 0.091 | 0.073 | 0.073 | 0.072 | 0.070 | 0.069 | 0.065 | 0.055 | 0.050 | 0.094 | 0.029 | 31% |
| **IC Strain Rate** | N | K | G | C | B | P | E | F | I | A | H | D | L | M | O | J | Q |  |  |  |
|  | 0.365 | 0.348 | 0.343 | 0.342 | 0.342 | 0.337 | 0.317 | 0.303 | 0.296 | 0.278 | 0.259 | 0.247 | 0.241 | 0.238 | 0.231 | 0.230 | 0.224 | 0.291 | 0.048 | 17% |
| **KTH** | N | P | C | B | G | K | E | F | Q | I | A | H | D | L | M | J | O |  |  |  |
|  | 0.291 | 0.281 | 0.279 | 0.273 | 0.271 | 0.264 | 0.244 | 0.227 | 0.219 | 0.218 | 0.206 | 0.185 | 0.182 | 0.178 | 0.171 | 0.166 | 0.163 | 0.225 | 0.044 | 20% |
| **Piper** | N | G | C | B | K | P | E | A | F | I | D | H | J | L | O | M | Q |  |  |  |
|  | 0.320 | 0.287 | 0.285 | 0.282 | 0.278 | 0.275 | 0.270 | 0.257 | 0.255 | 0.244 | 0.218 | 0.216 | 0.209 | 0.207 | 0.202 | 0.195 | 0.171 | 0.245 | 0.040 | 16% |
| **SIMon** | N | K | C | G | B | P | E | F | I | A | H | D | L | J | M | O | Q |  |  |  |
|  | 0.340 | 0.328 | 0.318 | 0.315 | 0.312 | 0.300 | 0.295 | 0.275 | 0.268 | 0.264 | 0.256 | 0.241 | 0.235 | 0.230 | 0.228 | 0.226 | 0.222 | 0.274 | 0.039 | 14% |
| **SIMon CSDM** | N | K | G | C | B | P | E | F | I | A | H | D | Q | L | J | O | M |  |  |  |
|  | 0.173 | 0.142 | 0.124 | 0.123 | 0.115 | 0.097 | 0.094 | 0.074 | 0.069 | 0.067 | 0.058 | 0.041 | 0.034 | 0.033 | 0.033 | 0.030 | 0.030 | 0.079 | 0.043 | 55% |
| **THUMS** | N | G | C | B | K | P | E | A | F | I | D | H | L | J | O | M | Q |  |  |  |
|  | 0.347 | 0.312 | 0.306 | 0.303 | 0.300 | 0.290 | 0.283 | 0.268 | 0.263 | 0.256 | 0.222 | 0.221 | 0.212 | 0.211 | 0.202 | 0.201 | 0.194 | 0.258 | 0.046 | 18% |
| **UCDBTM** | D | C | B | N | P | I | G | K | E | F | L | A | H | Q | M | J | O |  |  |  |
|  | 0.277 | 0.262 | 0.256 | 0.256 | 0.253 | 0.247 | 0.246 | 0.224 | 0.215 | 0.202 | 0.183 | 0.180 | 0.172 | 0.163 | 0.157 | 0.140 | 0.130 | 0.210 | 0.046 | 22% |
| **WHIM** | N | K | G | C | B | P | E | F | I | A | H | D | J | L | O | M | Q |  |  |  |
|  | 0.295 | 0.280 | 0.275 | 0.274 | 0.272 | 0.266 | 0.260 | 0.251 | 0.247 | 0.247 | 0.229 | 0.223 | 0.216 | 0.216 | 0.214 | 0.211 | 0.204 | 0.246 | 0.028 | 11% |

**Figure S3.** Ranking of helmets for Xrot loading condition for the different brain models and the associated peak values (if not specified, the peak value represents peak strain).

|  |  |  |  |  |  |  |  |  |  |  |  |  |  |  |  |  |  | **Average** | **SD** | **CV** |
| --- | --- | --- | --- | --- | --- | --- | --- | --- | --- | --- | --- | --- | --- | --- | --- | --- | --- | --- | --- | --- |
| **GHBMC** | G | P | B | K | A | C | F | J | I | N | E | O | L | H | D | M | Q |  |  |  |
|  | 0.375 | 0.370 | 0.342 | 0.342 | 0.337 | 0.329 | 0.323 | 0.321 | 0.314 | 0.312 | 0.309 | 0.308 | 0.283 | 0.276 | 0.265 | 0.254 | 0.230 | 0.311 | 0.038 | 12% |
| **GHBMC CSDM** | G | P | B | K | A | C | F | J | I | E | N | O | L | H | D | M | Q |  |  |  |
|  | 0.254 | 0.250 | 0.205 | 0.199 | 0.196 | 0.173 | 0.166 | 0.153 | 0.149 | 0.144 | 0.142 | 0.127 | 0.095 | 0.085 | 0.068 | 0.062 | 0.038 | 0.147 | 0.061 | 42% |
| **IC** | G | P | F | K | I | B | E | A | N | C | J | O | M | H | Q | L | D |  |  |  |
|  | 0.173 | 0.136 | 0.125 | 0.122 | 0.120 | 0.105 | 0.105 | 0.104 | 0.097 | 0.089 | 0.086 | 0.083 | 0.071 | 0.070 | 0.061 | 0.054 | 0.046 | 0.097 | 0.032 | 33% |
| **IC Strain Rate** | G | P | K | F | B | A | I | E | N | C | J | O | H | M | L | D | Q |  |  |  |
|  | 0.398 | 0.374 | 0.353 | 0.349 | 0.340 | 0.335 | 0.334 | 0.319 | 0.314 | 0.308 | 0.303 | 0.298 | 0.261 | 0.261 | 0.251 | 0.216 | 0.211 | 0.307 | 0.051 | 17% |
| **KTH** | G | P | K | F | B | A | I | C | N | J | E | O | L | H | M | D | Q |  |  |  |
|  | 0.411 | 0.391 | 0.365 | 0.356 | 0.350 | 0.342 | 0.336 | 0.331 | 0.330 | 0.321 | 0.320 | 0.319 | 0.278 | 0.272 | 0.264 | 0.247 | 0.221 | 0.321 | 0.049 | 15% |
| **Piper** | G | P | K | B | A | F | C | I | E | J | N | O | L | H | M | D | Q |  |  |  |
|  | 0.355 | 0.348 | 0.324 | 0.324 | 0.318 | 0.311 | 0.304 | 0.298 | 0.298 | 0.294 | 0.293 | 0.281 | 0.255 | 0.249 | 0.241 | 0.220 | 0.201 | 0.289 | 0.042 | 14% |
| **SIMon** | G | P | B | K | A | F | C | E | I | J | N | O | L | H | M | D | Q |  |  |  |
|  | 0.350 | 0.344 | 0.321 | 0.320 | 0.318 | 0.308 | 0.301 | 0.298 | 0.297 | 0.293 | 0.290 | 0.279 | 0.255 | 0.250 | 0.243 | 0.231 | 0.207 | 0.288 | 0.038 | 13% |
| **SIMon CSDM** | G | P | B | K | A | C | F | J | E | I | N | O | L | H | M | D | Q |  |  |  |
|  | 0.205 | 0.197 | 0.153 | 0.150 | 0.145 | 0.129 | 0.123 | 0.117 | 0.114 | 0.109 | 0.103 | 0.097 | 0.064 | 0.055 | 0.047 | 0.040 | 0.019 | 0.110 | 0.051 | 46% |
| **THUMS** | G | P | B | K | A | C | F | J | I | N | E | O | L | H | M | D | Q |  |  |  |
|  | 0.469 | 0.463 | 0.425 | 0.422 | 0.416 | 0.398 | 0.391 | 0.383 | 0.380 | 0.372 | 0.371 | 0.357 | 0.325 | 0.315 | 0.287 | 0.284 | 0.248 | 0.371 | 0.060 | 16% |
| **UCDBTM** | H | F | G | I | E | P | B | K | J | N | M | C | D | A | O | Q | L |  |  |  |
|  | 0.217 | 0.215 | 0.215 | 0.209 | 0.202 | 0.202 | 0.190 | 0.188 | 0.186 | 0.181 | 0.180 | 0.175 | 0.169 | 0.168 | 0.166 | 0.142 | 0.122 | 0.184 | 0.025 | 14% |
| **WHIM** | G | P | K | B | A | F | I | E | C | N | J | O | L | H | M | D | Q |  |  |  |
|  | 0.312 | 0.306 | 0.292 | 0.290 | 0.288 | 0.286 | 0.277 | 0.276 | 0.273 | 0.271 | 0.268 | 0.262 | 0.241 | 0.238 | 0.237 | 0.216 | 0.202 | 0.267 | 0.030 | 11% |

**Figure S4.** Ranking of helmets for Yrot loading condition for the different brain models and the associated peak values (if not specified, the peak value represents peak strain).

|  |  |  |  |  |  |  |  |  |  |  |  |  |  |  |  |  |  | **Average** | **SD** | **CV** |
| --- | --- | --- | --- | --- | --- | --- | --- | --- | --- | --- | --- | --- | --- | --- | --- | --- | --- | --- | --- | --- |
| **GHBMC** | P | K | N | B | A | I | L | J | C | F | M | G | E | O | H | D | Q |  |  |  |
|  | 0.543 | 0.541 | 0.534 | 0.532 | 0.530 | 0.530 | 0.528 | 0.526 | 0.520 | 0.514 | 0.511 | 0.507 | 0.506 | 0.501 | 0.501 | 0.485 | 0.469 | 0.516 | 0.019 | 4% |
| **GHBMC CSDM** | P | K | N | L | B | I | A | J | C | M | F | E | G | O | H | D | Q |  |  |  |
|  | 0.406 | 0.401 | 0.395 | 0.394 | 0.390 | 0.390 | 0.388 | 0.385 | 0.378 | 0.363 | 0.361 | 0.354 | 0.351 | 0.349 | 0.349 | 0.323 | 0.307 | 0.370 | 0.027 | 7% |
| **IC** | I | A | B | K | H | E | G | J | P | F | C | L | N | Q | M | O | D |  |  |  |
|  | 0.479 | 0.472 | 0.456 | 0.449 | 0.424 | 0.422 | 0.410 | 0.409 | 0.407 | 0.391 | 0.364 | 0.359 | 0.343 | 0.322 | 0.316 | 0.314 | 0.301 | 0.390 | 0.056 | 14% |
| **IC Strain Rate** | B | I | A | P | K | L | C | J | N | G | E | H | F | M | O | D | Q |  |  |  |
|  | 0.568 | 0.566 | 0.566 | 0.563 | 0.558 | 0.550 | 0.539 | 0.538 | 0.537 | 0.536 | 0.535 | 0.533 | 0.533 | 0.511 | 0.503 | 0.503 | 0.475 | 0.536 | 0.025 | 5% |
| **KTH** | K | I | B | A | P | J | N | L | F | G | C | M | E | H | O | D | Q |  |  |  |
|  | 0.442 | 0.439 | 0.429 | 0.429 | 0.429 | 0.425 | 0.422 | 0.418 | 0.414 | 0.413 | 0.411 | 0.410 | 0.408 | 0.404 | 0.402 | 0.401 | 0.391 | 0.417 | 0.013 | 3% |
| **Piper** | P | K | I | N | B | A | L | J | C | M | F | G | E | O | H | D | Q |  |  |  |
|  | 0.427 | 0.426 | 0.421 | 0.419 | 0.418 | 0.417 | 0.416 | 0.411 | 0.406 | 0.401 | 0.399 | 0.397 | 0.392 | 0.391 | 0.388 | 0.377 | 0.360 | 0.404 | 0.018 | 4% |
| **SIMon** | P | L | B | N | K | C | A | J | F | I | H | M | E | O | G | D | Q |  |  |  |
|  | 0.561 | 0.549 | 0.548 | 0.546 | 0.545 | 0.539 | 0.539 | 0.534 | 0.527 | 0.525 | 0.518 | 0.518 | 0.516 | 0.512 | 0.512 | 0.492 | 0.470 | 0.527 | 0.022 | 4% |
| **SIMon CSDM** | P | N | K | L | B | J | A | C | I | F | M | O | H | E | G | D | Q |  |  |  |
|  | 0.525 | 0.513 | 0.511 | 0.509 | 0.501 | 0.494 | 0.492 | 0.486 | 0.481 | 0.467 | 0.462 | 0.457 | 0.453 | 0.447 | 0.444 | 0.407 | 0.389 | 0.473 | 0.037 | 8% |
| **THUMS** | K | P | N | J | A | B | I | L | C | F | M | O | H | G | E | D | Q |  |  |  |
|  | 0.681 | 0.676 | 0.666 | 0.652 | 0.649 | 0.649 | 0.643 | 0.642 | 0.621 | 0.612 | 0.606 | 0.599 | 0.595 | 0.591 | 0.589 | 0.548 | 0.533 | 0.621 | 0.041 | 7% |
| **UCDBTM** | A | K | B | H | G | E | F | I | P | D | C | J | M | O | Q | L | N |  |  |  |
|  | 0.350 | 0.348 | 0.344 | 0.344 | 0.340 | 0.338 | 0.329 | 0.328 | 0.321 | 0.316 | 0.307 | 0.307 | 0.304 | 0.299 | 0.298 | 0.294 | 0.286 | 0.321 | 0.020 | 6% |
| **WHIM** | P | B | L | A | I | K | C | N | J | F | G | M | E | H | O | D | Q |  |  |  |
|  | 0.405 | 0.402 | 0.401 | 0.398 | 0.397 | 0.397 | 0.395 | 0.395 | 0.388 | 0.386 | 0.386 | 0.384 | 0.384 | 0.382 | 0.377 | 0.375 | 0.355 | 0.389 | 0.012 | 3% |

**Figure S5.** Ranking of helmets for Zrot loading condition for the different brain models and the associated peak values (if not specified, the peak value represents peak strain).

|  |  |  |  |  |  |  |  |  |  |  |  |  |  |  |  |  |  | **Average** | **SD** | **CV** |
| --- | --- | --- | --- | --- | --- | --- | --- | --- | --- | --- | --- | --- | --- | --- | --- | --- | --- | --- | --- | --- |
| **GHBMC** | P | K | G | N | B | C | A | I | F | E | J | L | O | H | M | D | Q |  |  |  |
|  | 0.384 | 0.380 | 0.379 | 0.377 | 0.374 | 0.366 | 0.364 | 0.355 | 0.352 | 0.349 | 0.346 | 0.333 | 0.332 | 0.325 | 0.317 | 0.314 | 0.301 | 0.350 | 0.025 | 7% |
| **GHBMC CSDM** | P | G | K | B | N | A | C | I | F | J | E | L | O | H | M | D | Q |  |  |  |
|  | 0.232 | 0.221 | 0.219 | 0.215 | 0.210 | 0.204 | 0.201 | 0.188 | 0.184 | 0.181 | 0.178 | 0.165 | 0.160 | 0.148 | 0.143 | 0.133 | 0.118 | 0.182 | 0.033 | 18% |
| **IC** | G | I | K | B | P | A | E | F | C | H | N | J | L | M | Q | O | D |  |  |  |
|  | 0.238 | 0.234 | 0.233 | 0.233 | 0.224 | 0.214 | 0.213 | 0.202 | 0.193 | 0.189 | 0.187 | 0.183 | 0.161 | 0.153 | 0.152 | 0.149 | 0.139 | 0.194 | 0.033 | 17% |
| **IC Strain Rate** | G | P | K | B | N | I | C | F | A | E | J | H | L | O | M | D | Q |  |  |  |
|  | 0.426 | 0.424 | 0.420 | 0.417 | 0.405 | 0.399 | 0.396 | 0.395 | 0.393 | 0.390 | 0.357 | 0.351 | 0.347 | 0.344 | 0.337 | 0.322 | 0.303 | 0.378 | 0.037 | 10% |
| **KTH** | P | G | K | B | N | C | F | I | A | E | J | O | L | H | M | Q | D |  |  |  |
|  | 0.367 | 0.365 | 0.357 | 0.351 | 0.348 | 0.340 | 0.332 | 0.331 | 0.326 | 0.324 | 0.304 | 0.294 | 0.291 | 0.287 | 0.282 | 0.277 | 0.277 | 0.321 | 0.031 | 10% |
| **Piper** | P | G | N | K | B | C | A | F | I | E | J | L | O | H | M | D | Q |  |  |  |
|  | 0.350 | 0.346 | 0.344 | 0.343 | 0.341 | 0.332 | 0.331 | 0.322 | 0.321 | 0.320 | 0.305 | 0.293 | 0.292 | 0.284 | 0.279 | 0.272 | 0.244 | 0.313 | 0.030 | 10% |
| **SIMon** | P | K | B | G | N | C | A | F | E | I | J | L | H | O | M | D | Q |  |  |  |
|  | 0.402 | 0.397 | 0.394 | 0.392 | 0.392 | 0.386 | 0.373 | 0.370 | 0.370 | 0.363 | 0.352 | 0.346 | 0.342 | 0.339 | 0.329 | 0.321 | 0.299 | 0.363 | 0.029 | 8% |
| **SIMon CSDM** | P | K | N | G | B | C | A | F | I | E | J | L | O | H | M | D | Q |  |  |  |
|  | 0.273 | 0.268 | 0.263 | 0.258 | 0.256 | 0.246 | 0.235 | 0.221 | 0.219 | 0.218 | 0.215 | 0.202 | 0.195 | 0.189 | 0.179 | 0.163 | 0.147 | 0.220 | 0.037 | 17% |
| **THUMS** | P | K | N | B | G | A | C | I | F | J | E | L | O | H | M | D | Q |  |  |  |
|  | 0.477 | 0.468 | 0.462 | 0.459 | 0.457 | 0.445 | 0.442 | 0.426 | 0.422 | 0.416 | 0.414 | 0.393 | 0.386 | 0.377 | 0.365 | 0.352 | 0.325 | 0.417 | 0.043 | 10% |
| **UCDBTM** | G | B | I | P | D | K | E | F | C | H | N | A | M | J | Q | L | O |  |  |  |
|  | 0.267 | 0.263 | 0.261 | 0.259 | 0.254 | 0.253 | 0.252 | 0.249 | 0.248 | 0.244 | 0.241 | 0.233 | 0.214 | 0.211 | 0.201 | 0.200 | 0.198 | 0.238 | 0.023 | 10% |
| **WHIM** | P | G | K | B | N | C | A | F | I | E | J | L | O | H | M | D | Q |  |  |  |
|  | 0.326 | 0.324 | 0.323 | 0.321 | 0.320 | 0.314 | 0.311 | 0.308 | 0.307 | 0.307 | 0.291 | 0.286 | 0.285 | 0.283 | 0.277 | 0.271 | 0.254 | 0.301 | 0.021 | 7% |

**Figure S6.** Ranking of helmets for the average of all three loading conditions for the different brain models and the associated peak values (if not specified, the peak value represents peak strain).

**Table S2.** The different thresholds for the star rating for the different brain models. A value below 25^th^ percentile value gave a 4-star rating, between 25^th^ and 50^th^ percentile value a 3-star rating, between 50^th^ and 75^th^ percentile value a 2-star rating, and above the 75^th^ percentile value a 1-star rating.

|  | **25th percentile** | **50th percentile** | **75th percentile** |
| --- | --- | --- | --- |
| **GHBMC** | 0.330 | 0.352 | 0.375 |
| **GHBMC CSDM** | 0.157 | 0.184 | 0.211 |
| **IC** | 0.159 | 0.193 | 0.226 |
| **IC Strain Rate** | 0.346 | 0.393 | 0.408 |
| **KTH** | 0.290 | 0.326 | 0.348 |
| **Piper** | 0.290 | 0.321 | 0.342 |
| **SIMon** | 0.341 | 0.370 | 0.392 |
| **SIMon CSDM** | 0.193 | 0.219 | 0.256 |
| **THUMS** | 0.384 | 0.422 | 0.458 |
| **UCDBTM** | 0.213 | 0.248 | 0.255 |
| **WHIM** | 0.284 | 0.307 | 0.321 |

**Table S3.** Evaluation of the brain models against different experiments with focus on intracranial response and middle to high rate.

|  | **Intracranial pressure** | | | | | **Displacement** | | | | | | | **Strain** | | | | | |
| --- | --- | --- | --- | --- | --- | --- | --- | --- | --- | --- | --- | --- | --- | --- | --- | --- | --- | --- |
|  | **Hardy et al.**^7,8^ | | | **Nahum et al.**^14^ | **Trosseille et al.**^16^ | **Hardy et al.**^7,8^ | | | **Alshareef et al.**^1,2^ | | | **Guettler et al.**^6^ | **Alshareef et al.**^1,2^ | | | **Zhou et al.**^21,22^ | | |
| **Rotation axis** | **X** | **Y** | **Z** | **Y** | **Y** | **X** | **Y** | **Z** | **X** | **Y** | **Z** | **Y** | **X** | **Y** | **Z** | **X** | **Y** | **Z** |
| **GHBMC**^5,13,18^ | Yes | Yes | Yes | Yes | Yes | Yes | Yes | Yes | Yes | Yes | Yes | No | No | No | No | No | No | No |
| **IC*** | No | No | No | No | No | No | No | No | Yes | Yes | No | No | No | No | No | No | No | No |
| **KTH**^5,10,11^ | Yes | Yes | Yes | Yes | No | Yes | Yes | Yes | No | No | No | No | No | No | No | No | No | No |
| **PIPER** ^12^ | No | No | No | No | No | Yes | Yes | Yes | No | No | No | No | No | No | No | No | No | No |
| **SIMon**^15^ | No | No | No | Yes | Yes | Yes | Yes | No | No | No | No | No | No | No | No | No | No | No |
| **THUMS**^3,5^ | Yes | Yes | Yes | Yes | Yes | Yes | Yes | Yes | No | No | No | No | No | No | No | No | No | No |
| **UCDTBM**^17^ | No | No | No | No | No | No | Yes | Yes | No | No | No | No | No | No | No | No | No | No |
| **WHIM**^19,20^****** | No | No | No | Yes | Yes | Yes | Yes | Yes | Yes | No | No | Yes | Yes | No | No | Yes | Yes | Yes |

*The evaluation of the IC model is presented below.
** The WHIM model has also been evaluated against volunteer experiments.

**Evaluation of IC model’s prediction of brain displacement against rotational cadaver experiments**

All the brain FE models in this study were validated against at least one cadaver experiment. However, the IC model is the only model that is yet to publish the validation. Hence, we briefly present the results of the validation of the IC model here.

Recent well-documented and open-access post-mortem human subject (PMHS) experiments allowed us to compare the model predictions of relative brain/skull deformations with those measured in controlled head rotations. In the experiments, a controlled rotational motion was applied to PMHS heads and the displacements of 24 receiving crystals inserted in the brain were measured with the sonomicrometry approach^1,2^. The initial positions of the receivers were measured at a resolution of 0.625mm. The key dimensions of the heads were measured and reported^1^.

We simulated four tests on two subjects (IDs: 846 and 900). The length, breadth and height of the IC FE model were scaled to match the dimensions of the subjects. Then, the centre of gravity was determined using the method explained in Alshareef et al.^2^ (briefly 8mm anterior to tragus on the Frankfurt plane and 25% of the distance between the Frankfurt and head vertex in the superior direction). A MATLAB code was developed to read the coordinates of the initial position of each receiver and find the nearest FE node. The maximum difference between the position of the receivers in the PMHS and corresponding nodes in the IC FE model was 1mm.

Coronal and sagittal motions with 40rad/s peak rotational velocity and 30ms duration were simulated for both subjects. In the simulations, the rigid skull of the model was loaded by applying the three components of the linear acceleration and three components of the rotational velocity measured during the experiments at the centre of gravity. The displacements of the receiver nodes were recorded with respect to the head axes fixed at the centre of gravity.

The x, y and z components of PMHS receiver displacements are overlaid onto the displacements of the corresponding nodes of the model in Figures S7-10, where the time history comparison for each receiver (R) is plotted separately for the first 90ms of the head motion. This time window was enough to capture more than two pulses of local brain displacement. Good agreement was observed between predictions and experiments for a number of receivers and axes.

To provide an objective measure of correlation between the predicted and experimental pulses the CORA analysis was performed. The implementation of the CORA analysis was based on Gehre et al.^4^ and the settings suggested in Giordano and Kleiven^5^ were adopted. The results shown in Figure S11 confirm that a majority of the displacement predictions have fair to excellent fidelity. However there are a few predictions that have marginal or unacceptable fidelity, which warrant further investigation beyond the scope of the current study. The mean CORA for all tests and axes range from 0.50 to 0.65 (Table S4), which indicates an overall fair fidelity of the predictions.


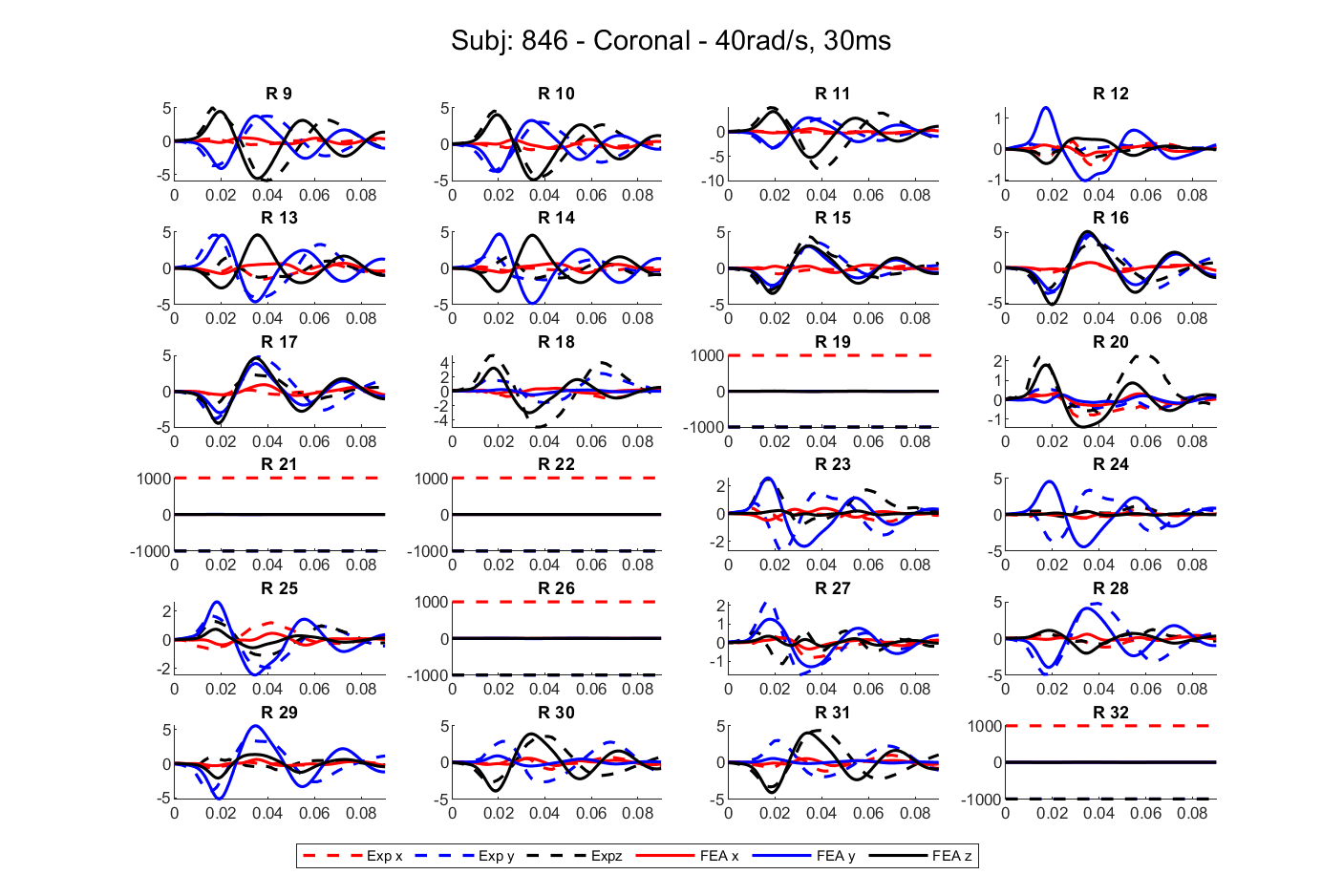


**Figure S7.** Relative brain/skull displacements at crystal receivers from the PMHS experiment (subject 846, coronal rotation) and predicted by IC model. The PMHS displacement data were not available for receivers R 19, 21, 22, 26 and 32.


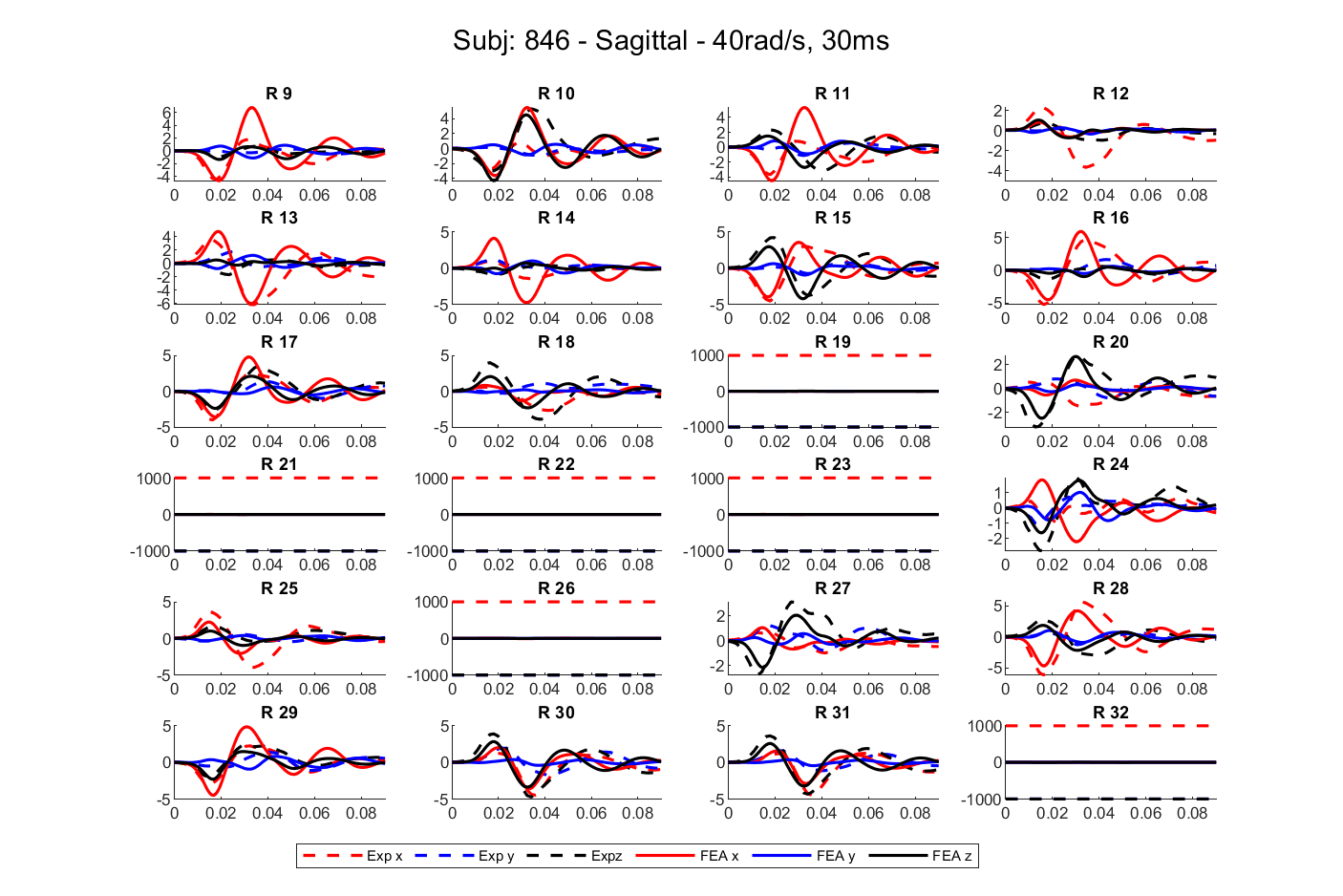


**Figure S8.** Relative brain/skull displacements at crystal receivers from the PMHS experiment (subject 846, sagittal rotation) and predicted by IC model. The PMHS displacement data were not available for receivers R 19, 21, 22, 26 and 32.


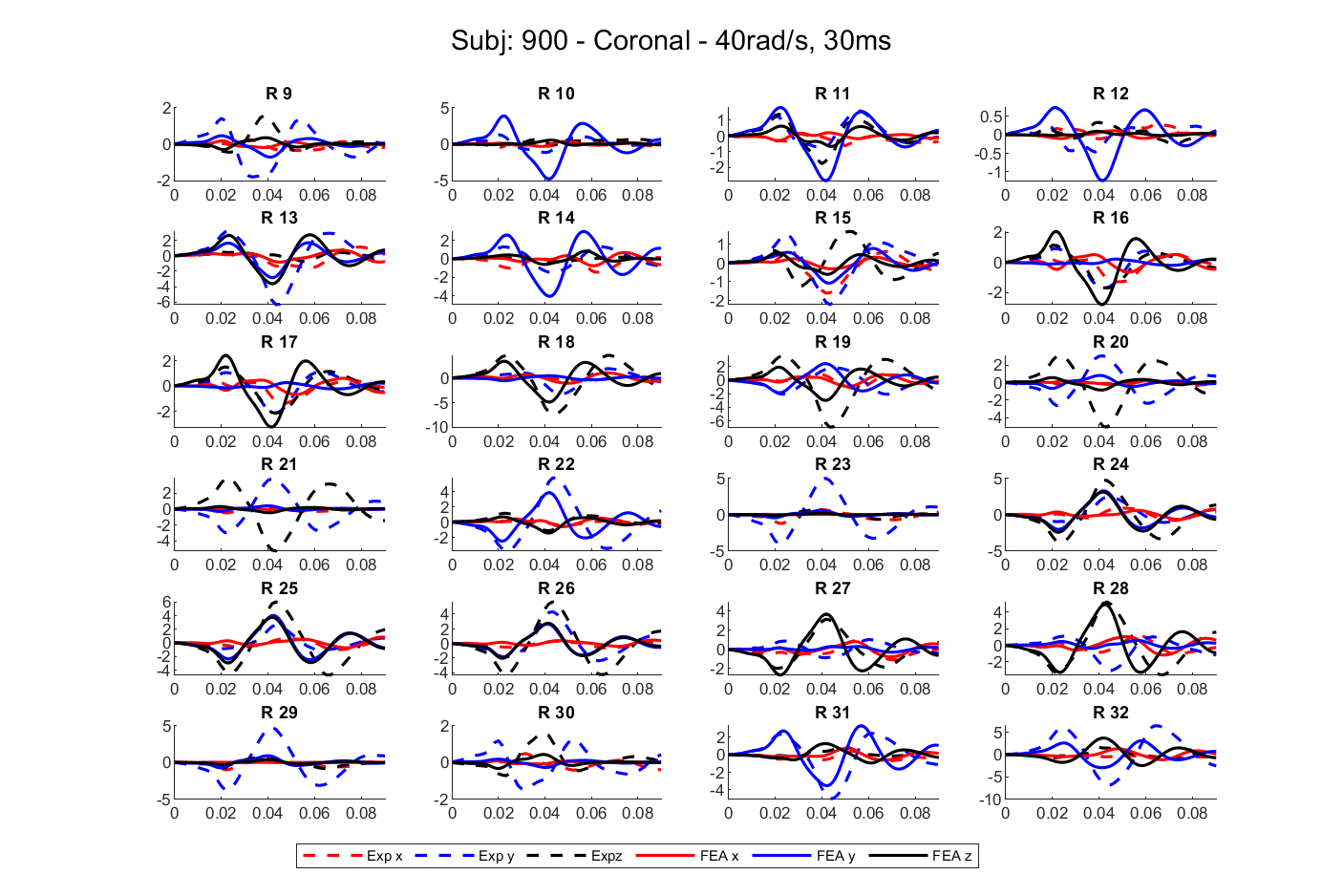


**Figure S9.** Relative brain/skull displacements at crystal receivers from the PMHS experiment (subject 900, coronal rotation) and predicted by IC model.


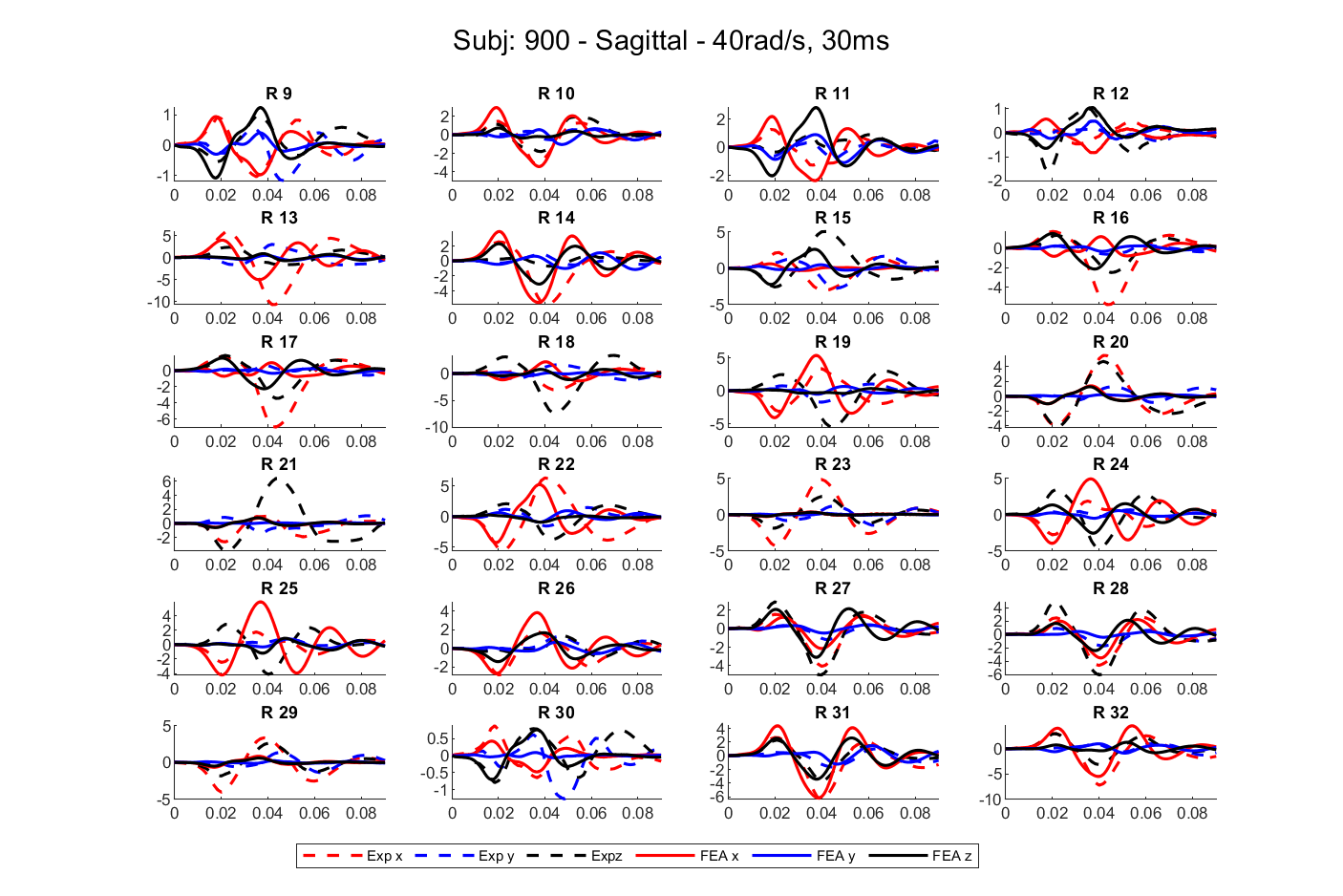


**Figure S10.** Relative brain/skull displacements at crystal receivers from the PMHS experiment (subject 900, sagittal rotation) and predicted by IC model.

| 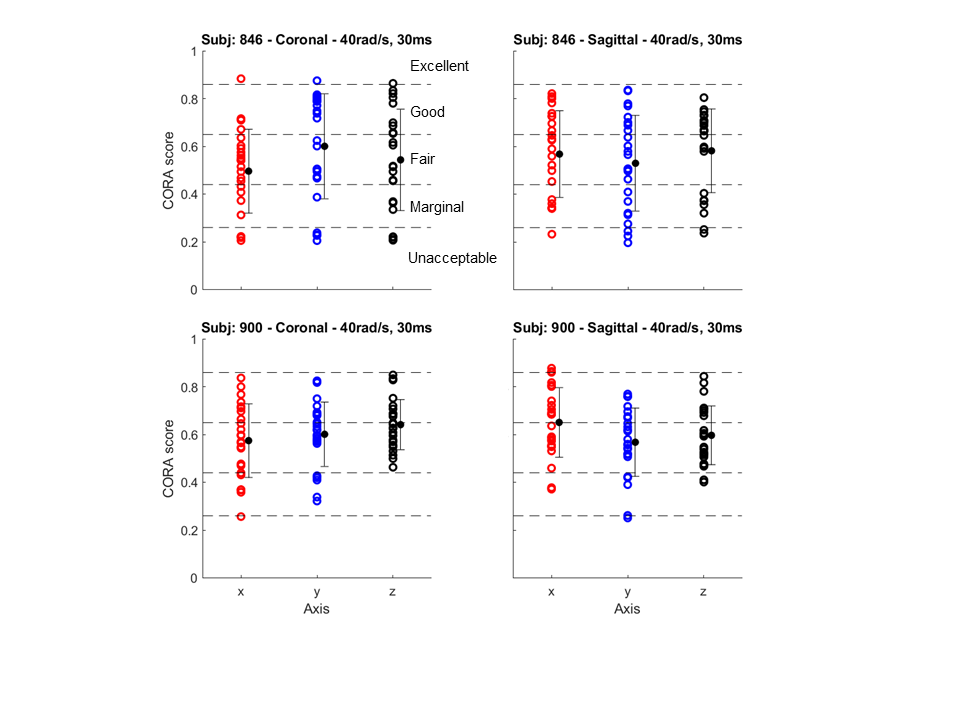 |
| --- |

**Figure S11.** CORA scores for the IC model predictions of the displacement of crystal receivers reported in rotational cadaver experiments. The broken lines indicate the borders of biofidelity classifications (unacceptable, marginal, fair, good and excellent) ) defined in ISO-TR 9790 (1999)^9^.

**Table S4.** The CORA scores for the predictions of the IC model of four rotational cadaver experiments with a motion of 40rad/s rotational velocity in 30ms.

| **Test no** | **Subject no** | **Plane of rotation** | **CORA**  **z axis** | **CORA**  **y axis** | **CORA**  **z axis** | **CORA overall** |
| --- | --- | --- | --- | --- | --- | --- |
| 12850 | 846 | Coronal | 0.50 | 0.60 | 0.54 | 0.55 |
| 12854 | 846 | Sagittal | 0.57 | 0.53 | 0.58 | 0.56 |
| 12874 | 900 | Coronal | 0.58 | 0.60 | 0.64 | 0.61 |
| 12878 | 900 | Sagittal | 0.65 | 0.57 | 0.60 | 0.61 |

# References

1. Alshareef, A., J. S. Giudice, J. L. Forman, D. F. Shedd, K. A. Reynier, T. Wu, S. Sochor, M. R. Sochor, R. S. Salzar, and M. B. Panzer. Biomechanics of the Human Brain During Dynamic Rotation of the Head. *Neurotrauma* , 2020.

2. Alshareef, A., J. S. Giudice, J. Forman, R. S. Salzar, and M. B. Panzer. A Novel Method for Quantifying Human In Situ Whole Brain Deformation under Rotational Loading Using Sonomicrometry. *J. Neurotrauma* 35:780– 789, 2017.

3. Atsumi, N., Y. Nakahira, and M. Iwamoto. Development and Validation of a Head/Brain FE Model and Investigation of Influential Factor on the Brain Response during Head Impact. *Int. J. Veh. Saf.* 9:1–23, 2016.

4. Gehre, C., H. Gades, and P. Wernicke. Objective Rating of Signals Using Test and Simulation Responses. , 2009.

5. Giordano, C., and S. Kleiven. Development of an Unbiased Validation Protocol to Assess the Biofidelity of Finite Element Head Models used in Prediction of Traumatic Brain Injury. *Stapp Car Crash J.* 60:363–471, 2016.

6. Guettler, A. J., R. Ramachandra, J. Bolte, and W. N. Hardy. Kinematics Response of the PMHS Brain to Rotational Loading of the Head: Development of Experimental Methods and Analysis of Preliminary Data. *SAE Tech. Pap.* 1–14, 2018.doi:10.4271/2018-01-0547

7. Hardy, W. N., C. D. Foster, M. J. Mason, K. H. Yang, A. King, and S. Tashman. Investigation of Head Injury Mechanisms Using Neutral Density Technology and High-Speed Biplanar X-ray. *Stapp Car Crash J.* 45:1–32, 2001.

8. Hardy, W. N., M. J. Mason, C. D. Foster, C. S. Shah, J. M. Kopacz, K. H. Yang, A. I. King, J. Bishop, M. Bey, W. Anderst, and S. Tashman. A Study of the Response of the Human Cadaver Head to Impact. *Stapp Car Crash J.* 51:17–80, 2007.

9. ISO/TR 9790. The International Organization for Standardization (ISO). Road vehicles—Anthropomorphic Side Impact Dummy—Lateral Impact Response Requirements to Assess the Biofidelity of the Dummy. , 1999.

10. Kleiven, S. Evaluation of Head Injury Criteria Using a Finite Element Model Validated against Experiments on Localized Brain Motion , Intracerebral Acceleration. *Int. J. Crashworthiness* 11:65–79, 2006.

11. Kleiven, S., and H. Von Holst. Consequences of Reduced Brain Volume Following Impact in Prediction of Subdural Hematoma Evaluated with Numerical Techniques. *Traffic Inj. Prev.* 3:303–310, 2002.

12. Li, X., and S. Kleiven. Improved Safety Standards Are needed to Better Protect Younger Children at Playgrounds. *Sci. Rep.* 8:1–12, 2018.

13. Mao, H., L. Zhang, B. Jiang, V. V Genthikatti, X. Jin, F. Zhu, R. Makwana, A. Gill, G. Jandir, A. Singh, and K. H. Yang. Development of a Finite Element Human Head Model Partially Validated with Thirty Five Experimental Cases. *J. Biomech. Eng.* 135:1–15, 2013.

14. Nahum, A. M., R. Smith, and C. C. Ward. Intracranial Pressure Dynamics During Head Impact. , 1977.

15. Takhounts, E. G., S. a Ridella, V. Hasija, R. E. Tannous, J. Q. Campbell, D. Malone, K. Danelson, J. Stitzel, S. Rowson, and S. Duma. Investigation of Traumatic Brain Injuries Using the Next Generation of Simulated Injury Monitor (SIMon) Finite Element Head Model. *Stapp Car Crash J.* 52:1–31, 2008.

16. Trosseille, X., C. Tarriére, F. Lavaste, F. Guillon, and A. Domont. Development of a F.E.M of the Human Head According to a Specific Test Protocol. , 1992.

17. Trotta, A., J. M. Clark, A. McGoldrick, M. D. Gilchrist, and A. N. Annaidh. Biofidelic Finite Element Modelling of Brain Trauma: Importance of the Scalp in Simulating Head Impact. *Int. J. Mech. Sci.* 173:105448, 2020.

18. Wu, T., A. Alshareef, J. S. Giudice, and M. B. Panzer. Explicit Modeling of White Matter Axonal Fiber Tracts in a Finite Element Brain Model. *Ann. Biomed. Eng.* 47:1908–1922, 2019.

19. Zhao, W., and S. Ji. Displacement- and Strain-Based Discrimination of Head Injury Models across a Wide Range of Blunt Conditions. *Ann. Biomed. Eng.* 48:1661–1677, 2020.

20. Zhao, W., S. Ruan, and S. Ji. Brain Pressure Responses in Translational Head Impact: A Dimensional Analysis and A Further Computational Study. *Biomech. Model. Mechanobiol.* 14:753–766, 2015.

21. Zhou, Z., X. Li, S. Kleiven, and W. N. Hardy. Brain Strain from Motion of Sparse Markers. *Stapp Car Crash J.* 63:1–27, 2019.

22. Zhou, Z., X. Li, S. Kleiven, C. S. Shah, and W. N. Hardy. A Reanalysis of Experimental Brain Strain Data: Implication for Finite Element Head Model Validation. *Stapp Car Crash J.* 62:293–318, 2018.
